# Information Retrieval from Photoplethysmographic Sensors: A Comprehensive Comparison of Practical Interpolation and Breath-Extraction Techniques at Different Sampling Rates

**DOI:** 10.3390/s22041428

**Published:** 2022-02-13

**Authors:** Pierluigi Reali, Riccardo Lolatto, Stefania Coelli, Gabriella Tartaglia, Anna Maria Bianchi

**Affiliations:** 1 Department of Electronics Information and Bioengineering, Politecnico di Milano, 20133 Milano, Italy; stefania.coelli@polimi.it (S.C.); gabriella.tartaglia@mail.polimi.it (G.T.); annamaria.bianchi@polimi.it (A.M.B.); 2 Department of Management, Economics and Industrial Engineering, Politecnico di Milano, 20133 Milano, Italy; riccardo.lolatto@mail.polimi.it

**Keywords:** photoplethysmography, inter-beat intervals, interpolation, heart rate variability, breath, sampling rate

## Abstract

The increasingly widespread diffusion of wearable devices makes possible the continuous monitoring of vital signs, such as heart rate (HR), heart rate variability (HRV), and breath signal. However, these devices usually do not record the “gold-standard” signals, namely the electrocardiography (ECG) and respiratory activity, but a single photoplethysmographic (PPG) signal, which can be exploited to estimate HR and respiratory activity. In addition, these devices employ low sampling rates to limit power consumption. Hence, proper methods should be adopted to compensate for the resulting increased discretization error, while diverse breath-extraction algorithms may be differently sensitive to PPG sampling rate. Here, we assessed the efficacy of parabola interpolation, cubic-spline, and linear regression methods to improve the accuracy of the inter-beat intervals (IBIs) extracted from PPG sampled at decreasing rates from 64 to 8 Hz. PPG-derived IBIs and HRV indices were compared with those extracted from a standard ECG. In addition, breath signals extracted from PPG using three different techniques were compared with the gold-standard signal from a thoracic belt. Signals were recorded from eight healthy volunteers during an experimental protocol comprising sitting and standing postures and a controlled respiration task. Parabola and cubic-spline interpolation significantly increased IBIs accuracy at 32, 16, and 8 Hz sampling rates. Concerning breath signal extraction, the method holding higher accuracy was based on PPG bandpass filtering. Our results support the efficacy of parabola and spline interpolations to improve the accuracy of the IBIs obtained from low-sampling rate PPG signals, and also indicate a robust method for breath signal extraction.

## 1. Introduction

In recent years, the increasing availability of wearable devices for mobile and smart healthcare monitoring, both in clinical (e.g., [1,2]) and wellness applications [3,4], has promoted the photoplethysmography (PPG) as a valid alternative to electrocardiography (ECG) for heart rate (HR) and heart rate variability (HRV) monitoring [5]. HRV features are established markers of the autonomic nervous system (ANS) activity. Specifically, heart beating is regulated by the action of the sympathovagal balance [6], and its monitoring is useful for the management of chronic cardiovascular disorders and for the identification and prevention of acute episodes [7,8]. At the same time, it can serve as daily monitoring for healthy subjects to assess stress levels, physical activity, sleep quality, or emotions [9,10,11].

Traditionally, HRV analysis is conducted using the ECG signal, from which the sequence of the RR intervals (RRI, i.e., the time distance between consecutive R-peaks) over time can be precisely derived. However, daily monitoring of HRV through ECG requires proper placement of the electrodes or the adoption of sensorized devices, such as smart shirts [12], whose long-time usage might be uncomfortable, while durability is limited to a certain number of washes [13]. On the contrary, PPG sensors mounted on wearable devices (such as wristbands or rings [14]) can detect blood volume changes in peripheral vessels caused by cardiac beats without the inconvenience of wearing electrodes. Hence, PPG can provide a convenient alternative to measure HRV (or pulse rate variability, PRV, as it has sometimes been named). In addition, like ECG [15], PPG signal also carries information about breathing activity, which modulates specific waveform characteristics (amplitude, frequency, and baseline wander). For this reason, such signal can also be exploited to derive respiratory measures of clinical interest [16], as well as a breath signal that can be used, for instance, to improve respiratory sinus arrhythmia (RSA) estimates from HRV by means of bivariate approaches [17,18,19,20] or for sleep apnea detection [21].

Given the notable amount of information that this signal can provide, many studies have focused on improving HRV parameters extraction from PPG (e.g., see [22,23,24]), and several others deal with the derivation of respiratory signal surrogates [21,25,26,27]. However, only a few studies have systematically investigated the quality of HRV and respiratory features derived from low-sampling-rate PPG signals [28,29,30]. PPG has a much lower frequency content (generally, lying within the range 0.01–20 Hz [31,32]) than ECG (mostly 0–70 Hz in healthy subjects, with the need to reach at least 150–300 Hz for accurate characterization of the QRS complex [33]); thus, the identification of the fiduciary points in time is intrinsically affected by higher errors.

In addition, the need for reducing power consumption in wearable devices, to increase battery life, brings to the adoption of low sampling rates. This operation has the following two main downsides: a further reduction in the PPG bandwidth and an increased sampling (or discretization) error. Both can substantially reduce the accuracy of fiduciary points detection, leading to biased computation of the inter beat intervals (IBIs) and inaccurate estimates of the derived HRV parameters [34,35]. In a recent study, Choi et al. [28] measured the PPG signal in resting state for 20 min and computed several time- and frequency-domain HRV indices from the original and subsampled PPGs. They reported no statistical differences for any of the examined HRV indices if the PPG sampling frequency was higher than 25 Hz. However, their study did not take into account different protocol conditions nor the effect of PPG interpolation, which has been suggested to improve the accuracy of the extracted IBIs [36,37]. Sun et al. [37] demonstrated the efficacy of cubic-spline interpolation in reducing the sampling error produced by PPG subsampling. Specifically, HRV indices computed from PPG sampled at 100, 50, and 20 Hz, and processed with cubic-spline interpolation, were comparable to those extracted from the original PPG signal (collected at 200 Hz). In another study, Béres and colleagues [30] analyzed PPG signals recorded from participants at rest and concluded that sampling frequencies of at least 20 Hz and 50 Hz were required, respectively, with and without interpolation, to achieve accurate measures of time-domain and Poincaré plot indices. To reduce the computational cost required by spline interpolation, Baek et al. [36] proposed the parabola approximation to interpolate PPG peaks and demonstrated its efficacy in PPG signals recorded at 20 Hz, compared to the spline approach. However, these studies examined the efficacy of the respective interpolation techniques considering either a few sampling frequencies or participants in resting-state only. Thus, the effect of different PPG interpolation strategies on the minimal adequate sampling rate for accurate HRV analysis still needs to be further investigated.

Moreover, low sampling rates can affect the quality of PPG-derived respiratory signals. For example, Charlton and colleagues [29] compared several respiratory signals extracted from PPG with a reference one, and found evident decreases in quality characterizing PPG signals below 16 Hz. Their assessment focused on the temporal correlation between PPG-derived and reference respiratory signals, and was conducted with participants at rest, in a supine position.

In this study, we analyzed beat-to-beat IBI estimates, and time- and frequency-domain HRV indices, extracted from low-resolution PPG signals (i.e., lowering the sampling rate from 64 Hz down to 8 Hz), during an experimental protocol comprising three different tasks (rest, stand, and controlled respiration). In fact, subjects’ position and administered tasks are known to influence the characteristics of the PPG waveform [38], possibly affecting the derived HRV indices. We assessed the efficacy of three interpolation techniques to compensate for the discretization error introduced by subsampling. Specifically, (1) we compared the performance of parabola approximation, spline interpolation, and an approach based on linear regression to evaluate the sampling frequency at which the use of interpolation becomes essential to improve HRV estimates; (2) we contrasted the accuracy achieved with the original PPG with the one of the subsampled interpolated PPG to understand in which conditions interpolation can effectively restore the accuracy of the original signal. The accuracy of PPG-derived IBIs and HRV indices was evaluated through the absolute difference with the HRV extracted from a concurrent ECG (gold-standard). We complemented this part of the study by analyzing the accuracy of two different strategies of fiduciary points detection. Furthermore, (3) we explored the possibility of obtaining breath rate information from PPG signals sampled at different frequencies to understand the minimal sampling rate that allows for the extraction of a reliable respiratory signal. In particular, we estimated the spectral coherence to compare the frequency content similarity of the surrogates extracted from PPG with the respiratory signal collected through a thoracic belt.

## 2. Materials and Methods

### 2.1. Data Collection and Processing

Data were collected at the PHEEL laboratory of Politecnico di Milano (Milano, Italy). Eight healthy volunteers (age 26.4 ± 2.3, 4 males and 4 females) were informed about the study and asked to sign a written consent before data acquisition. The study was approved by the university Ethics Committee. During the experiment, ECG, PPG, and a breathing signal were recorded. ECG was collected with two electrodes positioned under the collarbones and one slightly above the navel. PPG was obtained through a sensor placed on the second phalanx of the middle finger (left hand), and the reference respiratory signal was recorded with a thoracic belt. The PPG sensor we employed (BVP-Flex/Pro, Thought Technology, Montreal, Canada) uses an infra-red LED and measures the amount of light reflected by the skin, which varies with the blood volume present in the underlying vessels. All signals were collected at 256 Hz using the commercial polygraph ProComp Infiniti (Thought Technology, Montreal, Canada). During data acquisition, the preserved signal bandwidth was 0.05–120 Hz for the ECG; for the PPG and respiratory signal, it was 0–64 Hz. Participants were tested while performing a three-phased protocol for a total of 15 min, divided as follows: 0–5 min in a seated position (*Sit* phase); 5–10 min in a standing position (*Stand* phase); 10–15 min in which the subject is again in a sitting position and performs controlled respiration (*CR* phase), with cycles lasting 5 s (respiratory rate = 0.2 Hz).

Raw data were imported into MATLAB (The Mathworks Inc., Natick, MA, USA) and processed with custom scripts. Each subject’s RRIs were derived from the ECG by means of the Pan-Tompkins algorithm [39], which applies specific filters to reduce baseline wander, muscle artifacts, and other high-frequency noise components and performs R peak detection. After being processed with appropriate anti-aliasing finite impulse response (FIR) filters, the original PPG signals were subsampled at 64, 32, 16, and 8 Hz.

Since power consumption is a concern only when signals are collected with wearable devices, we focused on sampling rates up to 64 Hz, which is the frequency used for PPG recording by many current research-grade wearables, such as the E4 (Empatica, Boston, MA, USA) [40].

#### 2.1.1. PPG-Derived IBIs

Two beat detection algorithms were implemented for the PPG signal. A pseudo-code explanation of both algorithms is provided in Appendix A.

**Figure 1:**
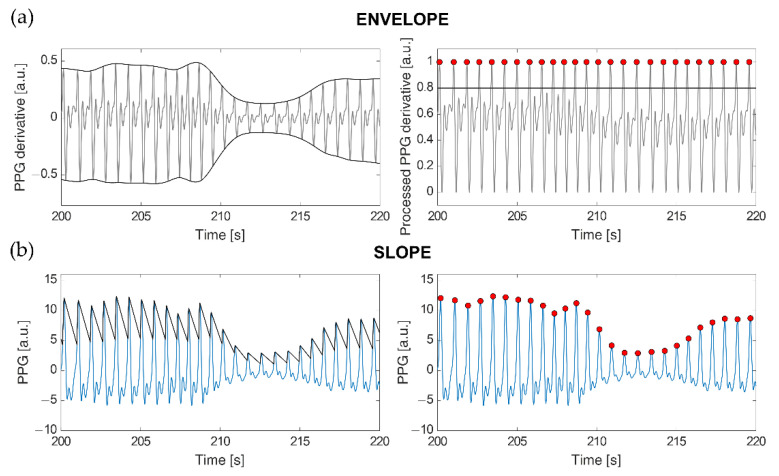
Application of the ENVELOPE (**a**) and SLOPE (**b**) beat detection methods to the respective signals of interest. (**a**) On the left, derivative of the PPG signal (grey) and relative superior and inferior envelopes (black); on the right, PPG derivative after subtraction of the inferior and superior envelopes (grey), threshold for peak detection (black line), and detected peaks (red circles). (**b**) On the left, the original PPG signal (light blue) and the computed adaptive threshold (black); on the right, detected peaks (red circles).

The ENVELOPE method (Figure 1a) implements a modified version of the algorithm described in [5]. Once the first derivative of the PPG signal is computed (*x*′(*t*)), the superior (*e_max_*(*t*)) and inferior (*e_min_*(*t*)) envelopes are calculated from its local maxima and local minima, respectively. These envelopes are used in the following min-max normalization to limit the signal amplitude between 0 and 1:

(1) $${x^{\prime }}_{norm}\left(t\right)=\frac{x^{\prime }\left(t\right)-e_{min}\left(t\right)}{e_{max}\left(t\right)-e_{min}\left(t\right)}\;$$

This method eliminates signal amplitude variations caused by the different protocol stages (e.g., in the Stand phase, signal amplitude is lower than during Sit) and fluctuations due to breath. Then, candidate heartbeats are identified as the local maxima exceeding an amplitude of 0.8. Local maxima of the PPG derivative correspond to the point of maximum slope on the ascending segments of the PPG signal. The 0.8 threshold was selected empirically, as it allowed us to detect almost all the true heartbeats, while avoiding slopes following dicrotic notches to be disguised as heartbeats. However, the local maxima detected with this threshold sometimes do not correspond to true heartbeats, as they can also arise from motion artifacts or dicrotic notches followed by particularly sharp peaks. In order to reduce such false positives, a procedure has been implemented that retains only those peaks producing the closest peak-to-peak distance to a weighted average calculated on the preceding beats ($\bar{IBI}$). Specifically, at each iteration, a valid peak (*j*) is selected as the one that minimizes the vector ***d***:

(2) $$d=\left|\frac{t-t_{i}}{\bar{IBI}}-1\right|=\left|\frac{\left[t_{1},t_{2},\ldots ,t_{N}\right]-t_{i}}{\bar{IBI}}-1\right|\;,\;i\in \left[1,N\right)\;$$

(3) $$j=\arg\underset{k\in \left(i,N\right]}{\min}d_{k}$$

(4) $$\bar{IBI}=0.8\;{\bar{IBI}}_{prev}+0.2\;\left(t_{j}-t_{i}\right)$$

In the equations, *t_i_* is the occurrence time of the currently processed peak, *t* is the vector of the occurrence times of all the detected peaks, and *N* is their total number. At each iteration, all the peaks included between *i* and the selected one (*j*), if any, are labeled as false positives; thus, during the next iteration, ***d*** is computed considering *i = j*. At the end of each iteration, $\bar{IBI}$ is updated as in (4), where ${\bar{IBI}}_{prev}$ refers to the value of $\bar{IBI}$ during the previous iteration, and *t_j_ − t_i_* is the IBI of the last accepted beat. For the first iteration, $\bar{IBI}$ initializes to the median IBI computed from all the *N* peaks.

The second approach we employed, SLOPE (Figure 1b), makes use of an adaptive threshold detection algorithm to compensate for PPG waveform variations caused by respiration and vasoconstriction or dilation [41]. A virtual threshold is firstly defined based on the original PPG waveform amplitude. To detect the following peak position, the value of this threshold is decreased over time till it intercepts the signal. Then, the threshold accompanies the signal waveform until it reaches the inflection point (i.e., the peak). After the adaptive threshold finds the peak, it decreases again with a slope parameter that is modified according to the last peak amplitude, the standard deviation of the PPG signal, and its sampling frequency [41]. Since PPG waveform could vary due to different factors, an adaptive refractory period was defined as 0.6 times the previous average pulse interval. If a new peak is found within the refractory period, that peak is ignored. Such a value was selected for our data as the one improving true heartbeat identifications while minimizing erroneous detections, especially due to dicrotic peaks.

These beat detection algorithms were applied to the original and subsampled PPG signals. First, the detected peaks were used to calculate the IBIs from the non-interpolated signals (hereafter ORIGINAL IBIs). Then, the position of the identified peaks was refined using the three strategies described below.

**Figure 2:**
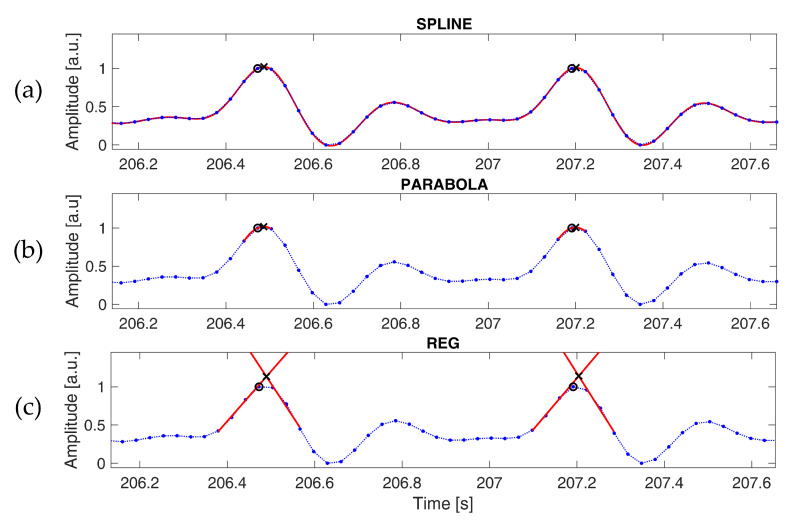
Application of the described interpolation strategies (**a**: SPLINE; **b**: PARABOLA; **c**: REG) to the peaks identified through the ENVELOPE method. The first derivative of a PPG signal sampled at 32 Hz is shown (blue dots). The following is in red: spline-interpolated first derivative (**a**), parabola approximation (**b**), regression curves estimated on the ascending and descending segments (**c**). The plots show the peaks detected on the subsampled signal (O) and those identified through interpolation (X).

Three different PPG interpolation techniques were tested to reduce the discretization error introduced by subsampling. The spline interpolation (SPLINE, Figure 2a) and parabola approximation (PARABOLA, Figure 2b) methods have been adopted and illustrated extensively in previous works [36,37]. In the former, samples around each peak are interpolated through cubic-spline, and the position of the original peak is replaced by the local maximum of the interpolated signal. In the latter, each peak is refined using the parabola defined by the three points closer to the detected peak; the original peak is replaced by the vertex of this parabola. The third method we tested (REG, Figure 2c) is based on linear regression. Each fiduciary point detected on the signal is replaced with the intersection of two distinct linear regression curves, as follows: the first curve is estimated on the ascending segment of the peak and the other one on the descending part. In this case, only the occurrence time is considered, as the amplitude is not a good estimation of the actual signal. Linear regression curves were computed after applying a lowpass filter with a cut-off frequency of 5 Hz, as commonly done in the literature, to reduce the effect of motion artifacts and high-frequency noise [42]. This operation systematically improved the accuracy of the estimated fiduciary points at every examined sampling rate. The number of points used for the regression was varied according to the sampling frequency in order to consider a time window of approximately 0.07 s, both for the ascending and descending segments.

The intersection of the two linear curves in the REG method, the occurrence time of the maximum of the spline interpolation and of the vertex of the parabola were calculated with a resolution of 1 ms.

#### 2.1.2. Extracted HRV Indices

Time- and frequency-domain features were computed from the extracted IBI series and reference HRV signals, considering each protocol phase separately. Time-domain indices included the average, standard deviation (SD), and root mean square of successive differences (RMSSD) of the IBIs and RRIs. As for the frequency-domain features, spectral powers in the low frequency (LF, 0.04–0.15 Hz) and high frequency (HF, 0.15–0.4 Hz) bands were computed in absolute and normalized units (n.u.), dividing each band power by the sum of LF and HF powers [43]. We adopted the autoregressive (AR) analysis [44,45] to estimate power spectral densities (PSDs) from the IBI and RRI series and the spectral decomposition method [46] to calculate LF and HF spectral powers. All the parameters were calculated in the central 4 min time interval of each protocol condition, thus considering at least 200 heartbeats for each segment; the first and last 30 s were excluded to avoid transients between consecutive protocol phases. Transients were removed to exclude non-stationary IBI and RRI epochs from the evaluation of the HRV indices of interest, which is particularly important for frequency-domain features.

#### 2.1.3. PPG-Derived Breath Signals

A more robust estimate of the RSA from HRV requires respiratory rate information. In fact, knowing the frequency content of respiration allows us to refine the frequency range for HF power calculation [47] and enables the quantification of cardiorespiratory interactions through bivariate models [17,18,19,20]. The respirogram, i.e., the breath signal sampled at the occurrence of each heartbeat, carries all the respiratory information needed for such analyses.

**Figure 3:**
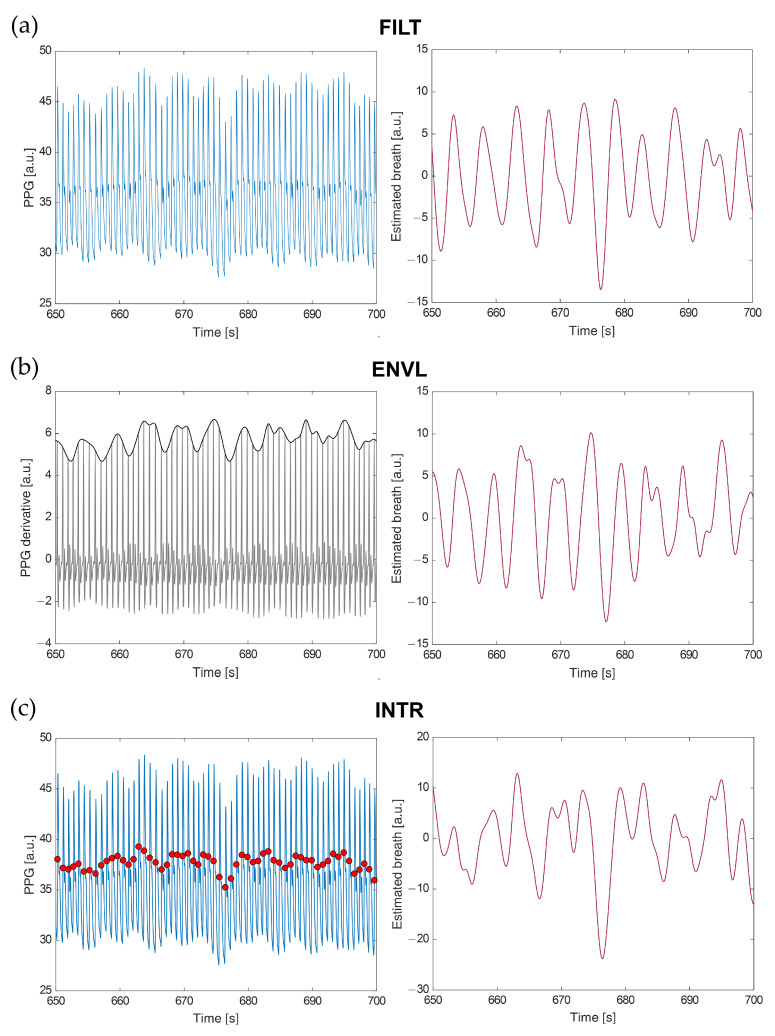
Application of the FILT (**a**), ENVL (**b**), and INTR (**c**) methods to the respective signals of interest. On the first column, input signals and main features are: (**a**) PPG signal (light blue); (**b**) derivative of the PPG signal (grey) and relative superior envelope (black); (**c**) PPG signal (light blue) and points of maximum slope (red). Second column: breath signals obtained with each method.

We examined three methods to extract breath signals from PPG, from which the respirograms were then derived. In the first one (FILT, Figure 3a), the PPG signal is filtered with a fourth-order Butterworth bandpass filter with cut-off frequencies of 0.13 and 0.48 Hz [48,49]; this method extracts respiratory-induced amplitude modulations in the PPG by removing frequencies outside the expected range of respiratory frequencies [50]. The second method builds on the assumption that respiration induces changes in arterial stiffness and intrathoracic pressure, affecting pulse wave velocity, blood flow, and blood velocity [51,52,53]. The first derivative of the PPG is largely adopted as an indicator of blood velocity [54]; hence, the fluctuations of its local maxima over time can be analyzed to extract respiratory information. Following this rationale, the second method comprises computing the first derivative of the PPG signal and extracting its superior envelope (ENVL, Figure 3b). Starting from the first derivative peaks, the third method estimates the cubic-spline interpolation curve from the corresponding points positioned on the original PPG signal (INTR, Figure 3c). This method performs feature-based extraction of a surrogate respiratory signal by tracking PPG amplitude variations of the maximum slope points during systole; cubic-spline interpolation is used to obtain a fixed-rate breath signal from such points [50]. To reduce noise on the breath signals derived through the last two strategies, noise frequencies were removed by filtering in the range 0.05–0.6 Hz (i.e., 3–36 breaths per minute) [21,29].

Respirograms were obtained from these surrogate breath signals by retaining only those samples corresponding to the R peaks of the ECG acquired simultaneously. The reference respirogram was derived in the same way from the respiratory signal collected through the thoracic belt. To assess frequency content similarity between the reference respirogram and the three PPG-extracted ones, we calculated the average magnitude-squared coherence around the modal respiratory rate of each phase. Specifically, the frequency band of interest was selected for each participant and protocol condition by descending from the peak of the reference spectrum until 20% of the maximum spectral power component was reached, as previously done in [21]. The magnitude-squared coherence was calculated as follows:

(5) $$C_{xy}\left(f\right)=\frac{{\left|P_{xy}\left(f\right)\right|}^{2}}{P_{xx}\left(f\right)\cdot P_{yy}\left(f\right)}\;\;\;\;,\;$$

where *P_xy_*(*f*) is the cross-spectrum between the reference respirogram (*x*) and the one derived from PPG with each of the three estimation methods (*y*); *P_xx_*(*f*) and *P_yy_*(*f*) represent the power spectral densities of the two respirograms. Coherence values range from 0 to 1, where the closer they are to 1, the higher is the coherence between *x* and *y* signals.

In one of the female participants, changing from the sitting to the standing posture caused the thoracic belt to move downwards (i.e., towards the abdomen), critically reducing the amplitude of the respiratory signal for the remainder of the session. Given the poor quality of the reference respiratory signal recorded in such a case, the analyses described in this section were carried out on 7 participants. This sample size was comparable to those adopted in several studies analyzing the accuracy of PPG- or ECG-derived breathing signals in healthy participants, as it can be inferred from the supplementary material of [50]. Moreover, the effect sizes computed for the pairwise comparisons discussed in Section 3.5 were always larger than 0.8, further proving low inter-subject variability in the indication of the preferable breath-extraction techniques.

### 2.2. Statistical Analysis

The principal statistical assessments presented in this paper are schematized in Table 1.

First, the performances of the examined beat detection algorithms (ENVELOPE and SLOPE) were evaluated considering the number of missing (false negatives, FN) and extra beats (false positives, FP) observed with each detection method with respect to the beats detected through the gold-standard (true positives, TP). In particular, we compared three percentage measures, namely the false negative rate (FNR), the false discovery rate (FDR), and the overall accuracy, defined as follows:

(6) $$\mathrm{FNR}=\frac{\mathrm{FN}}{\mathrm{TP}+\mathrm{FN}},\;\;\mathrm{FDR}=\frac{\mathrm{FP}}{\mathrm{TP}+\mathrm{FP}},\;\;\mathrm{Accuracy}=\frac{\mathrm{TP}}{\mathrm{TP}+\mathrm{FP}+\mathrm{FN}}\;.\;$$

TP + FN represents the total number of beats detected on the ECG signal; TP + FP is the number of beats detected by each method, either correctly or not; TP + FP + FN represents the total number of detected and non-detected beats.

Moreover, a Bland–Altman analysis was conducted to assess the stability of the fiduciary points detected through the ENVELOPE and SLOPE algorithms by comparing the corresponding IBIs with the RRIs computed on the ECG. In particular, we focused on the 95% limits of agreement (LOA, i.e., the average of the difference ±1.96 times the standard deviation of the difference) [55]. To improve table readability with minimal loss of accuracy, we reported 95% LOA as just ±1.96 times the standard deviation of the difference since the average of the difference was always below 0.3 ms.

We adopted the Bland–Altman analysis also to assess the beat-to-beat accuracy of the original and refined IBIs compared to the ECG-extracted RRIs. In addition, to assess statistical differences between the original IBIs and those refined using the three tested interpolation methods, regardless of the sign of such differences, we evaluated the absolute errors (AEs) of the IBI series with respect to the RRI series. AEs were also computed between the HRV indices extracted from the different IBI series and those derived from the RRIs. A Friedman’s test was then conducted for each protocol condition and sampling frequency to investigate the presence of statistically significant differences in the AEs calculated with the various PPG interpolation methods, followed by the appropriate Bonferroni-corrected post hoc tests.

To compare the accuracy of the three estimated respiratory signals, we performed multiple Friedman’s tests on the magnitude-squared coherence computed with each method with respect to the reference respirogram. Specifically, an independent test was conducted for each phase of the experimental protocol and sampling frequency of the PPG signal.

The significance level was set to α = 0.05 for all the statistical tests. In addition, non-parametric effect size measures, namely Cohen’s *r* for Wilcoxon’s signed-rank test [56] and Kendall’s *W* for Friedman’s test [57], were employed to assess the robustness of specific findings. Statistical analyses were conducted in MATLAB.

## 3. Results

### 3.1. Comparison of Beat Detection Approaches

Table 2 reports the FNR, FDR, and overall accuracy obtained with each beat detection method on all the subjects involved in the study, considering PPG signals sampled at 64 Hz. The ENVELOPE method resulted in the lowest FNR and was able to detect all the beats recognized on the ECG. On the contrary, the SLOPE approach minimized the FDR in all protocol phases except Stand. The ENVELOPE accuracy was higher during the Stand phase, pretty close to SLOPE in CR, and slightly lower during Sit, indicating better overall performances of the first method compared to the second. In particular, the evident FNR increase and accuracy decrease in the SLOPE method during Stand suggest reduced ability to detect fiduciary points with the lower-amplitude and less-clean PPG signals characterizing this phase. This tendency was even emphasized in PPGs subsampled at 8 Hz (Table S1 in the Supplementary Materials), for which the SLOPE method reported a dramatic increase in FNR in the Stand condition. The accuracy of the ENVELOPE method, instead, remained relatively stable across the different sampling rates and protocol phases.

Lowering the sampling rates from 64 to 8 Hz, we notice a detriment of the FNR and accuracy obtained with the ENVELOPE and SLOPE approaches (Table S1). Indeed, reducing the temporal resolution of the PPG waveform affected the ability of both algorithms to perform heartbeat detection. Nevertheless, the performances of ENVELOPE were always found superior to those of SLOPE for every sampling frequency.

To assess the stability of the fiduciary points sought by each method, we conducted a first beat-to-beat comparison of the IBIs measured through the ENVELOPE and SLOPE approaches with the RRIs computed on the ECG. Table 3 shows the results from this comparison, which was conducted using only those beats detected by both methods, in order to consider the same number of reference beats for the computation of the relevant statistics. With both the beat detection techniques, the average of the IBIs was the same as that computed on the RRIs, whereas the standard deviation was higher in all protocol phases. The 95% LOA values were lower for ENVELOPE than for SLOPE during the Stand condition and comparable during Sit and CR.

In summary, the IBIs computed with the ENVELOPE and SLOPE methods reported similar mean and standard deviation. However, the smaller 95% LOA, the lower FNR, and the higher beat detection accuracy shown in the Stand phase brought to the choice of considering only the ENVELOPE approach for the subsequent analyses. To further support this choice, we conducted a Wilcoxon’s signed-rank test between the AEs of IBI SLOPE and those of IBI ENVELOPE, both computed taking RRI as a reference and using PPGs sampled at 64 Hz. Test results show that the AEs related to the first pair (i.e., $\left|{\mathrm{IBI}}_{SLOPE}-\mathrm{RRI}\right|$) were larger than those calculated on the second (i.e., $\left|{\mathrm{IBI}}_{ENVELOPE}-\mathrm{RRI}\right|$) for the Stand condition (z = 1.904, *p* = 0.057), though not significantly, whereas they were very close during Sit (z = −0.489, *p* = 0.625) and CR conditions (z = 0.770, *p* = 0.441). These results support our choice of the ENVELOPE approach over the SLOPE one for further analyses.

### 3.2. Comparison of Interpolation Techniques: Beat-to-Beat

Table 4 shows the results of the beat-to-beat comparison performed between the RRIs, the original IBIs (derived through the ENVELOPE detection algorithm), and those extracted after applying the SPLINE, PARABOLA, and REG interpolation methods. Looking at the 95% LOA, we notice that SPLINE interpolation and PARABOLA approximation have IBI estimates closer to the RRIs than the original and REG IBIs. In particular, compared to the original IBIs, decreases in SPLINE and PARABOLA 95% LOA started to be visible already at 64 Hz and became more apparent from 32 Hz down. SPLINE and PARABOLA performances are similar at all the considered sampling frequencies, even if lower 95% LOA can be observed, in the Stand condition, at 16 and 8 Hz for the SPLINE method.

As regards the REG approach, the 95% LOA in Table 4 shows that IBIs more accurate than the original ones can be achieved at frequencies ranging from 64 to 32 Hz. Nevertheless, the accuracy achieved with REG was always comparable to or lower than that of SPLINE and PARABOLA approaches. Moreover, at 16 Hz, the IBIs computed through REG showed considerably lower accuracy than the original ones. Indeed, at such a low sampling frequency, the small number of samples available for each ascending and descending part of the PPG peaks limits the accuracy with which their morphology can be modeled using regression curves. Consequently, the REG method was not feasible with PPG sampled at 8 Hz.

**Figure 4:**
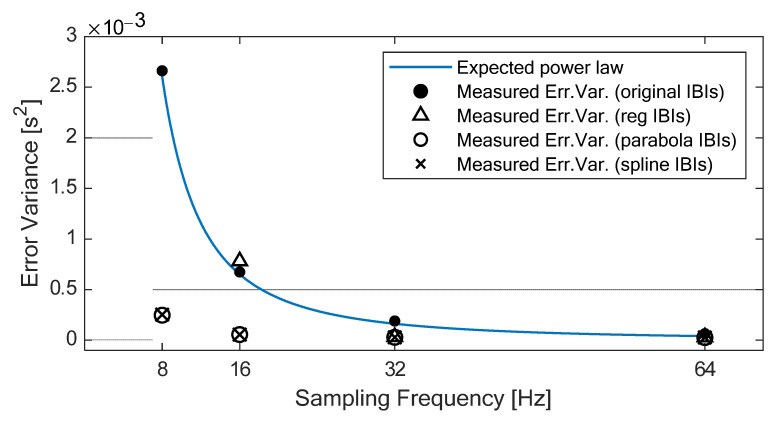
Comparison of the error variance measured on the IBIs with the theoretical one as a function of the sampling frequency. Without PPG interpolation (black points), the measured error variance resembles the expected power law. With interpolation (triangles, circles, and crosses), the error variance measured with the different methods almost overlaps from 64 to 32 Hz; at 16 Hz, an increase in error variance is observed with the REG method, whereas PARABOLA and SPLINE values remain overlapped.

According to Merri and colleagues [34], the error variance of the IBIs without interpolation should increase as the sampling period increases, following a power law relationship (i.e., ∆t^2^/6, where ∆t is the sampling period), as shown in Figure 4. In our experiment, the error variance of the original IBIs (which can be easily derived from the 95% LOA reported in Table 4) shows exactly this behavior, with high adherence to the expected power law relationship (R-squared = 0.999). On the contrary, the error variance of the IBIs derived from the interpolated PPGs shows slight variations across sampling rates down to 32 Hz for all three methods and down to 16 Hz for SPLINE and PARABOLA. In particular, down to 16 Hz, the use of either SPLINE or PARABOLA approaches allows achieving error variance (Figure 4) and 95% LOA (Table 4) comparable with the original IBIs computed from 64 Hz PPG.

**Figure 5:**
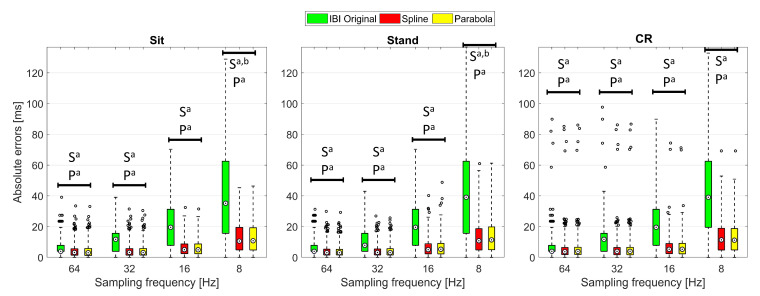
Boxplots of the beat-to-beat AEs computed with the original IBIs and with those obtained through SPLINE and PARABOLA interpolations. Significant differences (Bonferroni-corrected comparisons, *p* < 0.05) are highlighted between AEs within each sampling frequency and protocol condition. a: AEs related to SPLINE (S) or PARABOLA (P) are significantly different from the original; b: AEs related to SPLINE (S) are significantly different from PARABOLA.

To further investigate the advantages provided by the two best performing interpolation techniques, for each PPG sampling frequency and protocol phase, a Friedman’s test was conducted to compare the AEs related to the original IBIs with those computed through SPLINE and PARABOLA interpolations. The Friedman’s test reported *p* < 0.001 for all the sampling frequencies and protocol phases, indicating statistically significant AEs differences were always present among the evaluated methods. Yet, very different effect sizes (Cohen’s *r* [56], Table 5) were observed for post hoc tests (Figure 5). Specifically, significant differences between the original IBIs and those obtained through interpolation were found in post hoc tests conducted at 64 Hz, with effect sizes ranging from 0.397 to 0.471 depending on the specific protocol phase and interpolation method. Such effect sizes are typically interpreted as *medium* (i.e., *r* between 0.3 and 0.5 [56]), which suggests the application of interpolation methods already improves IBIs calculation starting from 64 Hz. The effect size of the difference between the original IBIs and those computed through interpolation becomes *large* (i.e., *r* higher than 0.5 [56]) at 32 Hz, indicating that the major advantages of using an interpolation approach, on a beat-to-beat basis, start at this sampling rate.

Regarding the comparison between SPLINE and PARABOLA approaches, a statistically significant difference with at least *small* (i.e., *r* between 0.1 and 0.3 [56]) effect size (Table 5) was found only in the post hoc test for the sampling rate of 8 Hz, limited to the Stand condition. This result statistically confirms that, for sampling rates higher than 8 Hz, SPLINE and PARABOLA provide IBIs with similar accuracies. However, at extremely low sampling rates (equal to 8 Hz, in our experiment), the SPLINE method might offer more accurate beat-to-beat measures, at least during the Stand condition, when PPG signal quality is generally lower than in the other protocol phases.

### 3.3. Comparison of Interpolation Techniques: HRV Indices

First, we explored HRV indices patterns per participant (supplementary Figures S1–S7) to assess if the variations imposed by the different sampling rates and interpolations were consistent. Since we found no notable disparities across subjects, subsequent analyses were run on the aggregated data.

**Figure 6:**
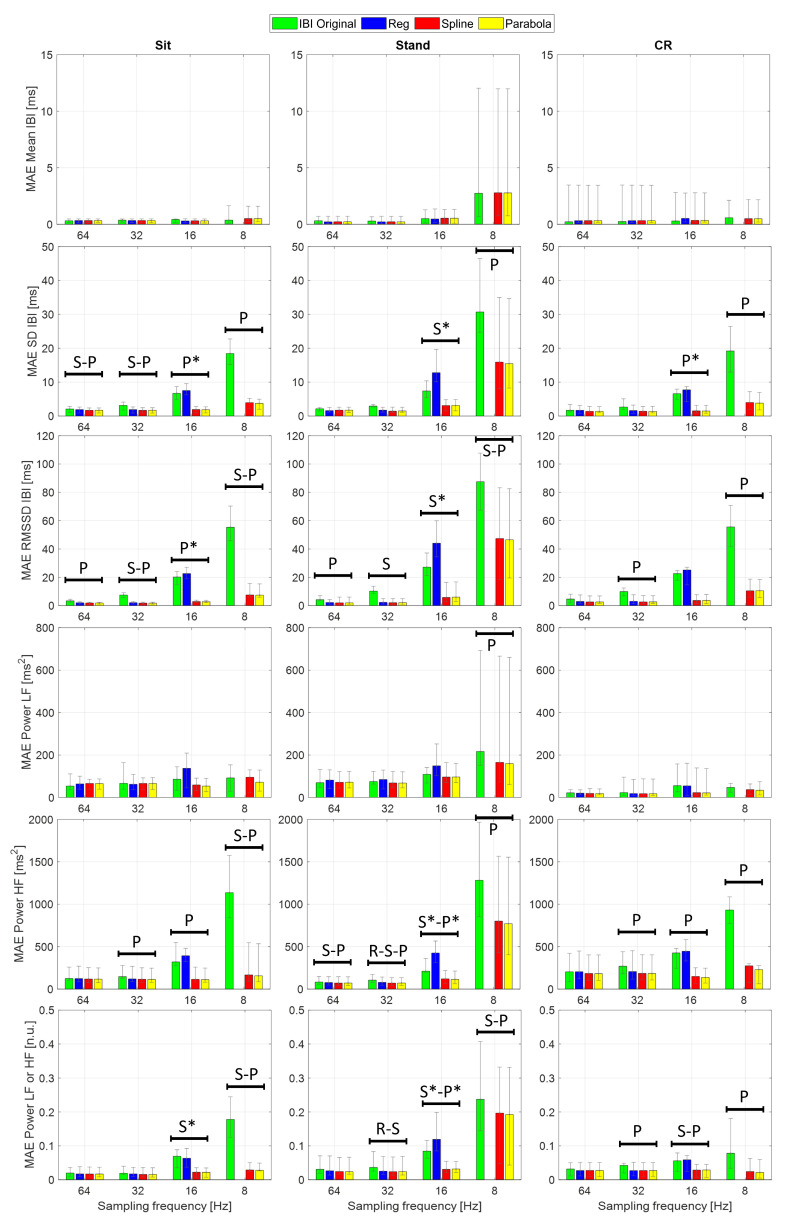
MAE (bars), upper, and lower quartiles (error bars) of the considered time- and frequency-domain features. R, S, and P indicate frequencies at which significant differences (Bonferroni-corrected, *p* < 0.05) were found between the original IBIs and REG (R), SPLINE (S), or PARABOLA (P) interpolations. All the reported differences show effect size (Cohen’s *r*) > 0.5. *: Significant difference revealed after excluding REG.

Figure 6 and the supplementary Tables S2 and S3 show the median absolute errors (MAEs) and error interquartile range of the examined HRV time- and frequency-domain parameters. Statistical analysis focused on the AEs to highlight accuracy differences among the considered IBI estimates, independent of the sign of the underlying variations. Specifically, one Friedman’s test for each combination of sampling frequency and protocol phase was conducted to assess whether statistically significant differences emerge in the HRV indices estimated using different PPG interpolation approaches. Table 6 reports the main statistics related to each test; as the AEs computed from the normalized LF and HF powers are equal by definition, so are the related test statistics. Apart from Mean IBI and Power LF, showing a single statistically significant difference (Friedman *p* < 0.05) in the Stand condition at 8 Hz, the other HRV indices reported significant differences among interpolation methods at several sampling rates and protocol phases. In particular, larger effect sizes (Kendall’s *W* [57]), thus more substantial differences, are visible at 16 and 8 Hz, especially in the Stand condition. Among time-domain parameters, Mean IBI was the only one showing little to no variation of the AEs among interpolation methods as the sampling rate decreased. This result finds confirmation in previous empirical work, such as [30], where interpolated and non-interpolated PPGs showed comparable accuracy in Mean IBI computation down to a sampling rate of 5 Hz. Further, it is supported by the theory since the discretization error is assumed as a zero-mean uniformly distributed white noise [34]. As regards frequency-domain indices, Power HF demonstrated higher sensitivity than Power LF to sampling rate reduction. Indeed, the HF frequency band tends to be more influenced by an increasing sampling error, and this finding was expected from the theory as well [34].

The AEs of several HRV indices significantly decreased after PPG interpolation, compared to the ones computed from the original IBIs, as confirmed by the Bonferroni-corrected post hoc tests (see Figure 6). For this analysis, given the limited number of observations, we reported only those differences that presented *p* < 0.05 in the post hoc test and *large* effect size (i.e., Cohen’s *r* above 0.5) to make our interpretation more robust.

First of all, SPLINE and PARABOLA interpolations allowed us to estimate HRV parameters that always improved or, at least, maintained the accuracy of the original IBIs. Considering these two approaches together, statistically significant AEs improvements started at 64 Hz sampling frequency. HRV indices benefiting from interpolation at such a high sampling rate were SD IBI, RMSSD, and Power HF.

Significant improvements became more frequent and consistent at 32 Hz, especially for RMSSD and Power HF, showing a significant reduction in AEs for SPLINE and PARABOLA in every protocol condition. At 8 Hz, all the examined HRV indices, except for Power LF (reporting a significant improvement only during Stand) and Mean IBI (showing no significant differences at all), exhibited significant decreases in the AEs with SPLINE and PARABOLA approaches, with a notable effect size (*r* > 0.8).

Regarding the comparison between SPLINE and PARABOLA, very similar AEs, hence accuracy, can be noted for both time- and frequency-domain parameters. However, the PARABOLA approximation produced the largest number of statistically significant decreases in AEs. In particular, at 8 Hz, this technique produced all the statistically significant AEs decreases also detected through the SPLINE approach and more. Overall, this result may indicate higher reliability of the PARABOLA approach in respect of SPLINE, showing higher consistency in the attribution of the lowest rank to the AEs of the former method compared to the latter.

The absence of a statistically significant difference at 16 Hz for those indices where the same was detected at the immediately preceding (32 Hz) and/or following (8 Hz) sampling rates could be due to the inclusion of the REG method in the comparison, acting as a confounding factor. In fact, the drop in accuracy that this method exhibited at 16 Hz for many indices prevents the highest rank from being consistently assigned to the AEs of the original IBIs, as generally happens at higher sampling frequencies. As a consequence, higher dispersion in ranks can be observed. In fact, in many of those cases, removing the REG method from the comparison, significant differences also emerged at 16 Hz between the AEs of the original and interpolated IBIs (Figure 6).

As in the beat-to-beat analysis (Section 3.2), the REG method exhibited comparable or reduced accuracy compared to the other interpolation techniques. At 16 Hz, in particular, this method showed significantly higher AEs than the original IBIs for several HRV indices.

Lastly, at 8 Hz, all the HRV indices showed larger MAEs during Stand compared to the other protocol conditions. The PPG collected in standing posture might include higher frequency components that were not preserved at such a low sampling rate, as will be illustrated in the subsequent section.

### 3.4. Comparison of the IBIs from 256 Hz PPG and Interpolated Ones

To investigate if SPLINE and PARABOLA interpolations effectively compensated for the discretization error introduced by subsampling, we contrasted the AEs calculated on the IBI series extracted from the original PPG (sampled at 256 Hz) with those computed on the IBIs derived from the interpolated PPG. We employed the original PPG for this evaluation to maximize the bandwidth and minimize the discretization error in the reference signal. Specifically, the following AEs were computed for each protocol phase:

(7) $$AE_{ORIGINAL,\;256\mathrm{Hz}}=\left|IBI_{ORIGINAL,256\mathrm{Hz}}-RRI\right|\;,$$

(8) $$AE_{SPLINE,FS}=\left|IBI_{SPLINE,FS}-RRI\right|\;,AE_{PARABOLA,FS}=\left|IBI_{PARABOLA,FS}-RRI\right|,\;$$

with *FS* decreasing from 64 Hz to 8 Hz.

The AEs of the original IBIs were pairwise compared with the AEs of the SPLINE and PARABOLA approaches using Wilcoxon’s signed-rank tests. Table 7 shows the *p*-values of each test and the related effect sizes (Cohen’s *r*). Significant increases in AEs (*p* < 0.05) showing at least *small* effect sizes (*r* > 0.1) were detected for both the interpolation methods at 16 and 8 Hz. This result indicates that the IBIs extracted from the 256 Hz PPGs and those derived from the interpolated ones are substantially equivalent down to a sampling rate of 32 Hz. Concerning lower rates, the statistically significant difference observed at 16 Hz was characterized by *medium* effect size, ranging from 0.306 to 0.383. Therefore, beat-to-beat IBIs extracted from 16 Hz PPGs processed with SPLINE and PARABOLA interpolations already appear quite different from those derived through 256 Hz PPGs. When PPG is subsampled at 8 Hz, the effect size markedly increases and becomes *large* for both the interpolation methods (0.665 ≤ *r* ≤ 0.688), suggesting that, at this sampling rate, the compensation provided by SPLINE and PARABOLA does not suffice to recover the information carried by the original signal. In fact, interpolation strategies only allow researchers to reduce the discretization error caused by subsampling, but they have no effects on the reduction in PPG bandwidth that comes with this operation. Overall, these results show that subsampling produces no substantial changes in the derived IBIs up to 32 Hz if SPLINE or PARABOLA interpolations are applied. Consequently, a PPG bandwidth of approximately 16 Hz is more than enough to achieve the highest accuracy in beat detection enabled by the PPG signal. In contrast, with PPG bandwidths of approximately 8 or 4 Hz—which relate to 16 and 8 Hz sampling rates, respectively—suboptimal accuracies are achieved, whose severity should be determined based on the specific application.

**Figure 7:**
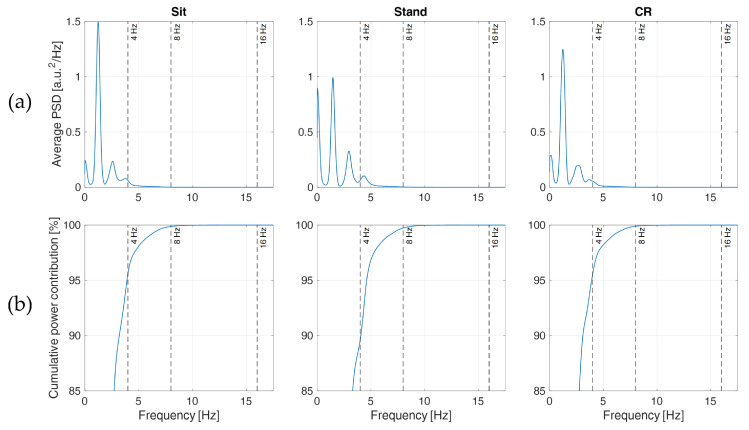
(**a**): average PSDs of the original PPGs (sampling frequency: 256 Hz, bandwidth: 0–64 Hz) collected from all the participants; (**b**): cumulative contribution of the different frequency components to total power (*y*-axis starts at 85% to highlight differences among phases). Columns represent different protocol phases. Each participant’s PSD was computed through Welch’s periodogram after standardizing the PPG signal with *z*-score to reduce inter-subject variability.

These considerations find support in Figure 7a, where the PSDs clearly show that most information content of the acquired PPGs lies below 8 Hz (see also, e.g., [28,41]), though less evident components are still present at higher frequencies, between 8 and 16 Hz. This explains why PPGs sampled at 32 Hz (preserving frequency components up to 16 Hz) and processed with SPLINE or PARABOLA interpolations produced IBIs that closely resembled those computed from the original PPG. In particular, Sit and CR phases reported cumulative power contributions (Figure 7b) of approximately 4.44%, 0.12%, and <0.01% for frequency components higher than 4, 8, and 16 Hz, respectively, implying a negligible loss of information with a sampling rate of 32 Hz. For the Stand phase, comparable values (0.27% and <0.01%) were reported for frequency components higher than 8 and 16 Hz, whereas a cumulative power contribution of 10.4% was found for frequency components higher than 4 Hz. This last finding might explain the MAE differences observed among protocol phases in Section 3.3. In fact, with a sampling rate of 8 Hz, the loss of information content due to subsampling is more than twice in the Stand condition compared to the rest of the protocol.

### 3.5. Comparison of the Respirograms Estimated from PPG

**Figure 8:**
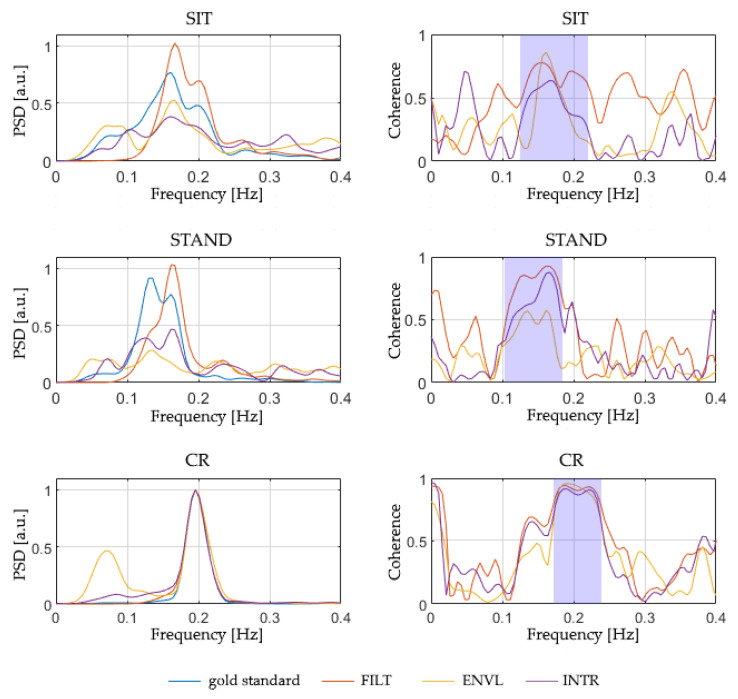
Example of PSD and magnitude-squared coherence computed for a single subject with the examined breathing signal estimation methods from a 64 Hz PPG signal. The shaded area in the coherence charts indicates the relevant frequency band selected for the participant.

Table 8 reports the magnitude-squared coherence between the reference respirogram and those estimated from PPG, averaged in the relevant band of the spectrum (Figure 8). This evaluation was performed separately for all the protocol phases and PPG sampling frequencies considered in the study. The coherence values shown in Table 8 are the median of the values obtained for all the subjects.

Although the ENVL method shows the highest values of magnitude-squared coherence for sampling rates of 64 and 32 Hz, a decline in its performance is observed at 16 Hz and 8 Hz. FILT, instead, shows a more stable performance, with similar values for all the considered sampling frequencies, revealing higher stability compared to the other methods. Finally, INTR systematically shows the lowest magnitude-squared coherence, demonstrating poor performances compared to the other two techniques.

To further investigate the advantages provided by each of the three methods and highlight possible differences between them, a Friedman’s test was conducted for each PPG sampling rate and protocol phase to compare their magnitude-squared coherences. Significant differences (*p* < 0.05) were detected mainly in the Sit and CR phases. The subsequent post hoc tests indicate that, concerning a sampling frequency of 64 Hz, a statistically significant difference (*p* < 0.05) is evident between the method that performs the best (ENVL) and the one that performs the worst (INTR); besides, a significant difference was detected between the latter and FILT, limited to the Sit condition. This result implicitly indicates that, at 64 Hz, ENVL and FILT perform in a similar way (since no significant difference arises between them). On the contrary, from 32 Hz below, statistical differences occurred between FILT and INTR methods, suggesting that FILT behaves better than INTR and, at the same time, FILT is comparable to ENVL, as well, due to the absence of statistical difference. In particular, concerning the PPGs sampled at 8 Hz, a significant difference occurs between FILT and ENVL during Stand, showing that, for lower sampling rates, ENVL performs poorly compared to FILT.

## 4. Discussion and Conclusions

In this paper, we have systematically studied the effect of decreasing sampling rate in PPG signal on time- and frequency-domain HRV parameters. We have tested the performances of two peaks detection methods (i.e., ENVELOPE and SLOPE) for the IBI time series construction and assessed the efficacy of three interpolation strategies (SPLINE, PARABOLA, and REG) in the refinement of peaks detection while decreasing the PPG sampling rate. In the same framework, we have explored the application of three simple algorithms to extract breath information from PPG (FILT, ENVL, and INTR), again with decreasing time resolution. To the best of our knowledge, the current study is the first to assess all the aspects above in the following three different conditions: sitting, standing, and controlled respiration. Because of this, we were able to identify the ENVELOPE detection method not only as the one performing better but also as the most stable across these protocol phases. Results also confirm the usefulness of interpolation procedures for peak detection when the sampling rate drops to 32 Hz, with similar performance for SPLINE and PARABOLA, while the REG method showed a lower performance. A consequent improvement was also observed in several HRV indices both in time and frequency domains. The beat-to-beat IBIs computed after SPLINE and PARABOLA interpolations were found to resemble those derived from the original 256 Hz PPG down to a sampling rate of 32 Hz, with moderate performance detriment observed at 16 Hz. In general, the accuracy improvements generated by SPLINE and PARABOLA approaches were consistent across the three protocol conditions. However, at 8 Hz sampling rate, the consequent PPG bandwidth reduction affected the accuracy of the computed HRV indices more in the Stand phase than in the other conditions. This finding suggests that our considerations should not be generalized to any task demand. Our results should be considered valid only for PPG collected during tasks requiring minimal (Sit phase) or mild (Stand and CR phases) physical effort. The effectiveness of PPG interpolation strategies and the minimum sampling rate required with higher physical loads should be further assessed.

Concerning the breathing signal estimation methods from PPG, the results indicate that ENVL is preferable at 64 Hz. Below that frequency, FILT should be preferred due to the higher stability of its performances across different sampling rates, especially considering Sit and CR phases. Thanks to its simplicity and the notable values of quadratic coherence achieved even with low sampling frequencies, FILT seems a convenient and accurate method to estimate respirograms from PPG.

In conclusion, our results suggest that PPG should not be collected with sampling rates lower than 16 Hz. PPG interpolation strategies are recommended with sampling rates below or equal to 32 Hz. However, at 16 Hz sampling frequency, none of the interpolations allow us to achieve the beat-to-beat accuracy of the 256 Hz PPG, indicating that a signal bandwidth of 8 Hz might already be too low for applications requiring highly reliable IBIs. SPLINE and PARABOLA methods provided comparable performances in all the considered conditions. Among the breath signal extraction techniques, ENVL performs better at 64 Hz sampling rate; FILT should be favored with lower sampling frequencies.

Given the increasingly widespread diffusion of PPG-based wearable devices for HRV monitoring, future extensions of this work are highly encouraged. In particular, since the rationale for choosing low sampling rates is to reduce power consumption, the additional power required by compensating techniques should be evaluated to ensure an actual reduction compared with the use of higher rates. Alternatively, strategies that do not require real-time processing, such as the interpolation methods we examined, may be offloaded to a separate device (e.g., a smartphone) to extend battery life in wearable PPG devices.

Further studies should validate our findings using PPG signals extracted from different body sites (e.g., PPG wristbands, rings, arm straps, or ankle straps) [58,59], possibly in various conditions, and considering a larger cohort of participants. In fact, site- and device-specific differences in performance can be expected for PPG interpolation and breathing signal extraction techniques.

## Figures and Tables

**Table 1:** Main statistical evaluations reported in the Results section. Each assessment was conducted for every sampling rate and protocol condition. The number of participants considered for each evaluation is reported.

Assessment	Performances of ENVELOPE and SLOPE Algorithms for Beat Detection	Beat-to-Beat Accuracy of the IBIs Derived from the Interpolated PPGs	Accuracy of the HRV Indices Computed from the Interpolated PPGs	Comparison of the IBIs Extracted from the Original and Interpolated PPGs	Accuracy of the Respirograms Estimated from PPG
**Aim**	To select the best beat detection strategy for the subsequent analyses	To analyze differences across interpolation methods on a beat-to-beat basis	To compare the HRV indices computed with different interpolation methods	To investigate at which sampling rates interpolation allows us to recover the IBI accuracy of the original PPGs	To indicate the most robust method for respirogram estimation from PPG
**Assessment methods**	FNR, FDR, and accuracy metrics; Bland–Altman; AEs testing	Bland–Altman; error variance; AEs testing	AEs testing	AEs testing; Power Spectral Density plots	Magnitude-squared coherence
**Subjects No.**	8	8	8	8	7
**Section**	3.1	3.2	3.3	3.4	3.5

**Table 2:** FNR, FDR, and accuracy observed with each method, grouped by protocol phases. Assessment performed with PPG signals sampled at 64 Hz.

	FNR (%)		FDR (%)		Accuracy (%)	
	SLOPE	ENVELOPE	SLOPE	ENVELOPE	SLOPE	ENVELOPE
Sit	0.17	0	0.04	0.33	99.79	99.67
Stand	0.46	0	0.07	0.04	99.47	99.97
CR	0.29	0	0.04	0.29	99.67	99.71

**Table 3:** Mean and standard deviation of the IBIs derived through ENVELOPE and SLOPE approaches; 95% limits of agreement (LOA) calculated between RRIs and each series of IBIs. Assessment performed with PPG signals sampled at 64 Hz.

		RRI	IBI SLOPE	IBI ENVELOPE
Sit	Mean ± SD (ms)	795.0 ± 90.3	795.0 ± 91.4	795.0 ± 91.9
	95% LOA (ms)	-	±15.4	±15.6
Stand	Mean ± SD (ms)	681.4 ± 67.8	681.4 ± 69.2	681.4 ± 69.4
	95% LOA (ms)	-	±17.0	±15.9
CR	Mean ± SD (ms)	797.8 ± 98.9	797.8 ± 99.9	797.8 ± 100.5
	95% LOA (ms)	-	±17.1	±16.7

**Table 4:** Mean and standard deviation of the RRIs and of the IBIs directly computed after beat detection (ORIGINAL) or refined through REG, SPLINE, and PARABOLA approaches; 95% LOA computed between the RRIs and each series of IBIs. The best performances in terms of 95% LOA are highlighted (green) for each sampling rate and protocol phase. The REG method was not appliable at 8 Hz.

				IBI			
			RRI	ORIGINAL	REG	SPLINE	PARABOLA
**FS = 64 Hz**	Sit	Mean ± SD (ms)	794.7 ± 90.8	794.8 ± 92.4	794.8 ± 92.3	794.8 ± 92.2	794.8 ± 92.2
		95% LOA (ms)	-	±15.8	±10.8	±10.2	±10.2
	Stand	Mean ± SD (ms)	681.3 ± 67.6	681.3 ± 69.3	681.3 ± 69.1	681.3 ± 68.9	681.3 ± 69.0
		95% LOA (ms)	-	±16.0	±11.1	±9.6	±9.6
	CR	Mean ± SD (ms)	797.2 ± 99.3	797.3 ± 100.7	797.3 ± 100.6	797.3 ± 100.5	797.3 ± 100.5
		95% LOA (ms)	-	±17.9	±13.3	±12.8	±12.8
**FS = 32 Hz**	Sit	Mean ± SD (ms)	794.9 ± 91.0	794.9 ± 93.3	795.0 ± 92.5	794.9 ± 92.5	794.9 ± 92.4
		95% LOA (ms)	-	±27.0	±11.3	±10.4	±10.5
	Stand	Mean ± SD (ms)	681.3 ± 67.6	681.3 ± 70.2	681.3 ± 69.1	681.3 ± 69.0	681.3 ± 69.0
		95% LOA (ms)	-	±26.9	±11.5	±9.8	±9.9
	CR	Mean ± SD (ms)	797.5 ± 99.7	797.5 ± 101.9	797.6 ± 101.1	797.6 ± 101.0	797.6 ± 101.0
		95% LOA (ms)	-	±28.0	±13.6	±12.8	±12.8
**FS = 16 Hz**	Sit	Mean ± SD (ms)	794.9 ± 91.0	794.9 ± 95.8	795.0 ± 96.5	794.9 ± 92.5	794.9 ± 92.5
		95% LOA (ms)	-	±50.9	±54.8	±14.6	±14.6
	Stand	Mean ± SD (ms)	681.5 ± 67.6	681.5 ± 73.3	681.5 ± 78.1	681.5 ± 69.1	681.5 ± 69.1
		95% LOA (ms)	-	±50.7	±71.7	±14.5	±15.4
	CR	Mean ± SD (ms)	797.9 ± 100.8	798.0 ± 105.1	798.0 ± 105.8	798.0 ± 102.1	798.0 ± 102.1
		95% LOA (ms)	-	±52.1	±54.1	±15.7	±15.7
**FS = 8 Hz**	Sit	Mean ± SD (ms)	796.7 ± 93.9	796.8 ± 107.6	-	796.8 ± 95.8	796.8 ± 95.6
		95% LOA (ms)	-	±101.1	-	±31.2	±30.8
	Stand	Mean ± SD (ms)	685.3 ± 65.8	685.6 ± 83.3	-	685.5 ± 68.4	685.5 ± 68.4
		95% LOA (ms)	-	±99.3	-	±31.4	±32.6
	CR	Mean ± SD (ms)	798.3 ± 100.7	798.3 ± 114.1	-	798.4 ± 102.5	798.4 ± 102.4
		95% LOA (ms)	-	±102.6	-	±30.6	±30.7

**Table 5:** Effect sizes (Cohen’s *r*) of the investigated differences. ORIG: beat-to-beat AEs of the original IBIs; SPL and PAR: beat-to-beat AEs of the IBIs derived from PPG signals interpolated through SPLINE and PARABOLA approaches, respectively.

	Sit			Stand			CR		
FS	r _ORIG–SPL_	r _ORIG–PAR_	r _PAR–SPL_	r _ORIG–SPL_	r _ORIG–PAR_	r _PAR–SPL_	r _ORIG–SPL_	r _ORIG–PAR_	r _PAR–SPL_
**64 Hz**	0.449	0.451	−0.019	0.470	0.471	−0.002	0.397	0.400	−0.021
**32 Hz**	0.698	0.701	0.029	0.700	0.698	0.057	0.660	0.663	0.010
**16 Hz**	0.808	0.811	−0.023	0.793	0.797	0.105	0.796	0.799	0.003
**8 Hz**	0.841	0.843	−0.061	0.830	0.831	0.219	0.840	0.844	0.035

**Table 6:** Comparison of the HRV indices estimated with the different PPG interpolation techniques. *p*-values and effect sizes (Kendall’s *W*) of Friedman’s tests conducted on the AEs of the considered HRV indices for each PPG sampling frequency (FS) and protocol phase. Test degrees of freedom were (3, 21) for all the FS except 8 Hz (2, 14) due to the inapplicability of REG at this FS.

			Mean IBI	SD IBI	RMSSD	Power LF	Power HF	Power LF (n.u.)	Power HF (n.u.)
**FS = 64 Hz**	Sit	*p*-value	0.795	0.002 **	0.012 *	0.415	0.041 *	0.047 *	0.047 *
		W	0.043	0.606	0.456	0.119	0.344	0.331	0.331
	Stand	*p*-value	0.861	0.092	0.041 *	0.861	0.005 **	0.036 *	0.036 *
		W	0.031	0.269	0.344	0.031	0.531	0.356	0.356
	CR	*p*-value	0.736	0.041 *	0.199	0.583	0.440	0.272	0.272
		W	0.053	0.344	0.194	0.081	0.112	0.163	0.163
**FS = 32 Hz**	Sit	*p*-value	0.920	0.019 *	0.012 *	0.682	0.029 *	0.050	0.050
		W	0.021	0.413	0.456	0.062	0.375	0.325	0.325
	Stand	*p*-value	0.717	0.041 *	0.034 *	0.415	0.006 **	0.001 **	0.001 **
		W	0.056	0.344	0.362	0.119	0.512	0.656	0.656
	CR	*p*-value	0.927	0.241	0.034 *	0.615	0.034 *	0.018 *	0.018 *
		W	0.019	0.175	0.362	0.075	0.362	0.419	0.419
**FS = 16 Hz**	Sit	*p*-value	0.868	0.004 **	0.004 **	0.583	0.006 **	0.011 *	0.011 *
		W	0.030	0.562	0.562	0.081	0.512	0.462	0.462
	Stand	*p*-value	0.930	<0.001 ***	<0.001 ***	0.199	<0.001 ***	<0.001 ***	<0.001 ***
		W	0.019	0.775	0.825	0.194	0.900	0.900	0.900
	CR	*p*-value	0.811	0.011 *	0.013 *	0.369	<0.001 ***	0.002 **	0.002 **
		W	0.040	0.462	0.450	0.131	0.850	0.619	0.619
**FS = 8 Hz**	Sit	*p*-value	0.197	<0.001 ***	0.002 **	0.325	0.002 **	0.002 **	0.002 **
		W	0.203	1.000	0.766	0.141	0.812	0.750	0.750
	Stand	*p*-value	0.030 *	0.001 **	0.002 **	0.021 *	0.001 **	0.002 **	0.002 **
		W	0.438	0.891	0.766	0.484	0.891	0.812	0.812
	CR	*p*-value	0.095	0.021 *	0.021 *	0.607	0.011 *	0.011 *	0.011 *
		W	0.294	0.484	0.484	0.062	0.562	0.562	0.562

The number of asterisks highlights test significance: * *p* < 0.05; ** *p* < 0.01; *** *p* < 0.001.

**Table 7:** *p*-value (*p*) of the Wilcoxon’s signed-rank test conducted on SPLINE vs. ORIGINAL and PARABOLA vs. ORIGINAL pairs of beat-to-beat AEs, for each PPG sampling frequency (FS) and protocol phase (in this case, ORIGINAL IBIs are derived from PPG signals sampled at 256 Hz). Additionally, the effect size of each pairwise comparison (Cohen’s *r*) is reported.

		FS = 64 Hz		FS = 32 Hz		FS = 16 Hz		FS = 8 Hz	
		*p*	*r*	*p*	*r*	*p*	*r*	*p*	*r*
Sit	SPLINE	0.088	0.035	0.633	0.010	<0.001	0.383	<0.001	0.688
	PARABOLA	0.022	0.047	0.454	0.015	<0.001	0.374	<0.001	0.686
Stand	SPLINE	<0.001	0.095	0.005	0.054	<0.001	0.351	<0.001	0.685
	PARABOLA	<0.001	0.097	0.044	0.038	<0.001	0.362	<0.001	0.696
CR	SPLINE	0.215	0.025	0.883	0.003	<0.001	0.310	<0.001	0.665
	PARABOLA	0.062	0.038	0.675	0.009	<0.001	0.306	<0.001	0.672

**Table 8:** Median and interquartile range of the magnitude-squared coherence between the original breathing signal and each of the three estimation methods, grouped by protocol phases.

	SIT			STAND			CR		
FS	FILT	ENVL	INTR	FILT	ENVL	INTR	FILT	ENVL	INTR
**64 Hz**	0.74 ± 0.18	0.80 ± 0.26	0.48 ± 0.12 ^a,b^	0.55 ± 0.47	0.70 ± 0.29	0.57 ± 0.38	0.80 ± 0.17	0.86 ± 0.12	0.72 ± 0.24 ^b^
**32 Hz**	0.74 ± 0.18	0.77 ± 0.27	0.38 ± 0.12 ^b^	0.54 ± 0.46	0.68 ± 0.29	0.48 ± 0.27	0.80 ± 0.17	0.82 ± 0.11	0.57 ± 0.15 ^a^
**16 Hz**	0.74 ± 0.18	0.61 ± 0.30	0.32 ± 0.25 ^a^	0.55 ± 0.46	0.56 ± 0.21	0.48 ± 0.18	0.81 ± 0.17	0.72 ± 0.12	0.43 ± 0.34 ^a^
**8 Hz**	0.74 ± 0.18	0.34 ± 0.16	0.14 ± 0.15 ^a^	0.54 ± 0.47	0.38 ± 0.20 ^a^	0.49 ± 0.25	0.80 ± 0.17	0.65 ± 0.16	0.37 ± 0.39 ^a^

^a.^ Significantly different from FILT (Bonferroni-corrected comparisons, *p* < 0.05). ^b.^ Significantly different from ENVL (Bonferroni-corrected comparisons, *p* < 0.05). All the reported differences show effect size (Cohen’s *r*) > 0.8.

## Data Availability

Access to raw data is restricted due to privacy limitations. Aggregated data is available from the corresponding author upon reasonable request.

## Supplementary Materials

The following are available online at https://www.mdpi.com/article/10.3390/s22041428/s1, Figures S1–S7: Line charts of the time- and frequency-domain HRV indices estimated for each subject with the considered PPG interpolation methods, grouped by protocol phase and sampling frequency. Table S1: FNR, FDR and accuracy observed with each method, assessed with PPG sampling rates ranging from 32 to 8 Hz. Tables S2 and S3: Median absolute error (MAE) and error interquartile range (IQR) of the time- and frequency-domain HRV indices estimated for each PPG sampling frequency, interpolation method, and protocol condition.

## Appendix A

Table A1 illustrates a pseudo-code explanation of the ENVELOPE and SLOPE algorithms. Detailed description of the steps involved in the SLOPE algorithm can be found in [41].

**Table A1:** Pseudo-code of the ENVELOPE and SLOPE beat detection algorithms. Bold type in variable names indicates arrays.

ENVELOPE	SLOPE
# PPG PROCESSING Compute: ***x’*(*t*)** = first derivative of the PPG signal ***e_min_*(*t*)** = inferior envelope of ***x’*(*t*)** ***e_max_*(*t*)** = superior envelope of ***x’*(*t*)** ***x’_norm_*(*t*)** = min-max normalization of ***x’*(*t*)** as in Equation (4) # HEARTBEAT DETECTION Define: ***th_ENV_*** = threshold for the identification of heartbeats from ***x’_norm_*(*t*)**; set to 0.8 Find: ***t_peaks_*** = occurrence time of the local maxima of ***x’_norm_*(*t*)** exceeding ***th_ENV_*** # FALSE POSITIVES REDUCTION Define: $\bar{IBI}$= median peak-to-peak distance between all the elements in **t_peaks_** ***N*** = number of peaks in **t_peaks_** ***i*** = 1 While ***i*** < ***N*** Take the occurrence time of the ***i***-th peak (***t_i_***) Calculate the difference between ***t_i_*** and the occurrence time of all the other elements in ***t_peaks_***: ***t_i_*− *t***_1_, ***t_i_* − *t*_2_**, …, ***t_i_* − *t_N_*** Compare these differences to $\bar{IBI}$ by using Equation (2), from which ***d*** = [***d*_1_**, ***d*_2_**, …, ***d_N_***] is obtained For each ***i*** < ***k*** ≤ ***N***, find the lowest ***d_k_*** and define: ***j*** = ***k***-th peak that minimizes ***d*** Discard all the peaks included between ***i*** and ***j***-1 from ***t_peaks_***, if any, as they are false positives Update $\bar{IBI}$ as in Equation (4) and set ***i* = *j*** for the next iteration End # IBI CALCULATION Compute: **IBI_ENV_** = difference between consecutive elements of the remaining occurrence times in ***t_peaks_***	# PPG PROCESSING Define: ***x*(*t*)** = PPG signal ***N*** = number of samples in ***x*(*t*)** # HEARTBEAT DETECTION Define: ***RP*** = refractory period; initialized to 0.6 s ***RP_change_*** = multiplier for RP updates; set to 0.6 ***slope*** = slope coefficient for the adaptive threshold; initialized to 0.2*max(***x*(*t*)**) ***t_init_*** = occurrence time of the first increasing segment found in ***x*(*t*)** ***th*(*t*)** = adaptive threshold, initialized to ***x*(*t_init_*)** ***i*** = sample of ***x*(*t*)** corresponding to ***t_init_*** While ***i*** < ***N*** Increase ***i*** until a peak is found on ***x*(*t*)**; define ***j*** as the sample of ***x*(*t*)** corresponding to this peak Add ***t_j_*** (occurrence time of the peak) to the array of the peaks found so far (***t_peaks_***) Make ***th*(*t*)** = ***x*(*t*)** until the ***j***-th sample is reached Update the ***slope*** parameter as shown in Equation (2) of [41] Linearly decrease ***th*(*t*)** starting from the peak found on ***x*(*t*)**, using the updated ***slope*** When ***th*(*t*)** intersects ***x*(*t*)**: If RP has passed Proceed to step 7 Else Continue to linearly decrease ***th*(*t*)** and repeat this check at the next point of intersection End Compute the average IBI ($\bar{IBI}$) from the ***t_peaks_*** found so far and update RP for the next iteration: RP = RP_change_ ∗ $\bar{IBI}$ Update ***i*** to the sample where ***th*(*t*)** intersected ***x*(*t*)** and proceed with the next iteration End # IBI CALCULATION Compute: **IBI_SLOPE_** = difference between consecutive elements of ***t_peaks_***
