# Supplementary material for: Information Retrieval from Photoplethysmographic Sensors: A Comprehensive Comparison of Practical Interpolation and Breath-Extraction Techniques at Different Sampling Rates
Source: Sensors (Basel). 2022 Feb 13;22(4):1428. doi: 10.3390/s22041428 (PMC8877143; doi:10.3390/s22041428)
Supplement: Supplementary file 1 [file sensors-22-01428-s001.zip › sensors-1557580-supplementary-done.pdf]

sensors

Article

Information Retrieval from Photoplethysmographic Sensors: A Comprehensive Comparison of Practical Interpolation and Breath-Extraction Techniques at Different Sampling Rates

Pierluigi Reali [1,*], Riccardo Lolatto [2], Stefania Coelli [1], Gabriella Tartaglia [1] and Anna Maria Bianchi [1]

1 Department of Electronics Information and Bioengineering, Politecnico di Milano, 20133 Milano, Italy; stefania.coelli@polimi.it (S.C.); gabriella.tartaglia@mail.polimi.it (G.T.); annamaria.bianchi@polimi.it (A.M.B.)
2 Department of Management, Economics and Industrial Engineering, Politecnico di Milano, 20133 Milano, Italy; riccardo.lolatto@mail.polimi.it
* Correspondence: pierluigi.reali@polimi.it

Supplementary Material

1. Supplementary Figures

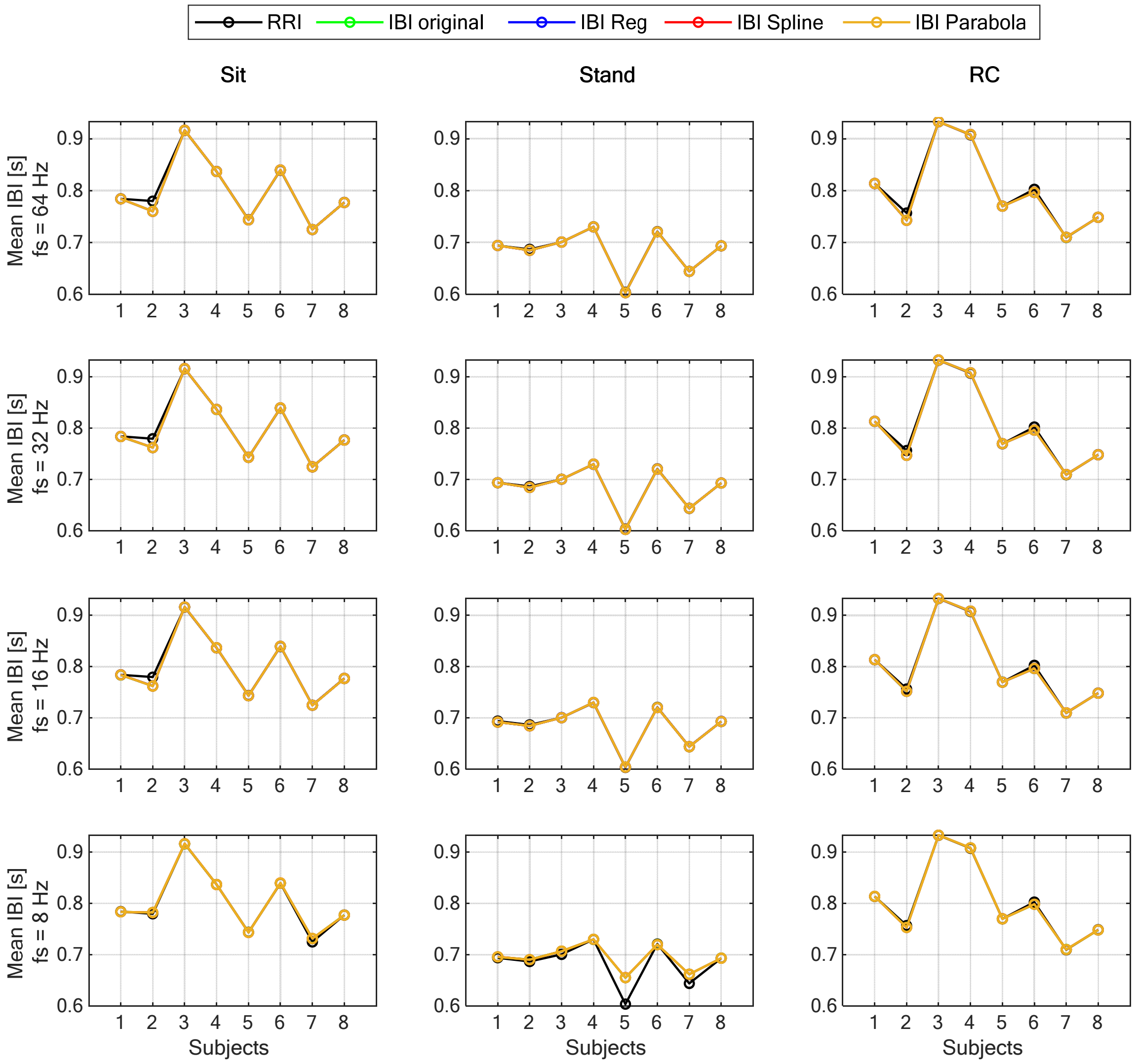

Figure S1. Line charts of the mean RRI and IBI estimated for each subject with the considered PPG interpolation methods, grouped by protocol phase and sampling frequency. As explained in the paper, the REG method could not be applied to PPG signals subsampled at 8 Hz; thus, none of the HRV indices was computed for the REG approach at this frequency.

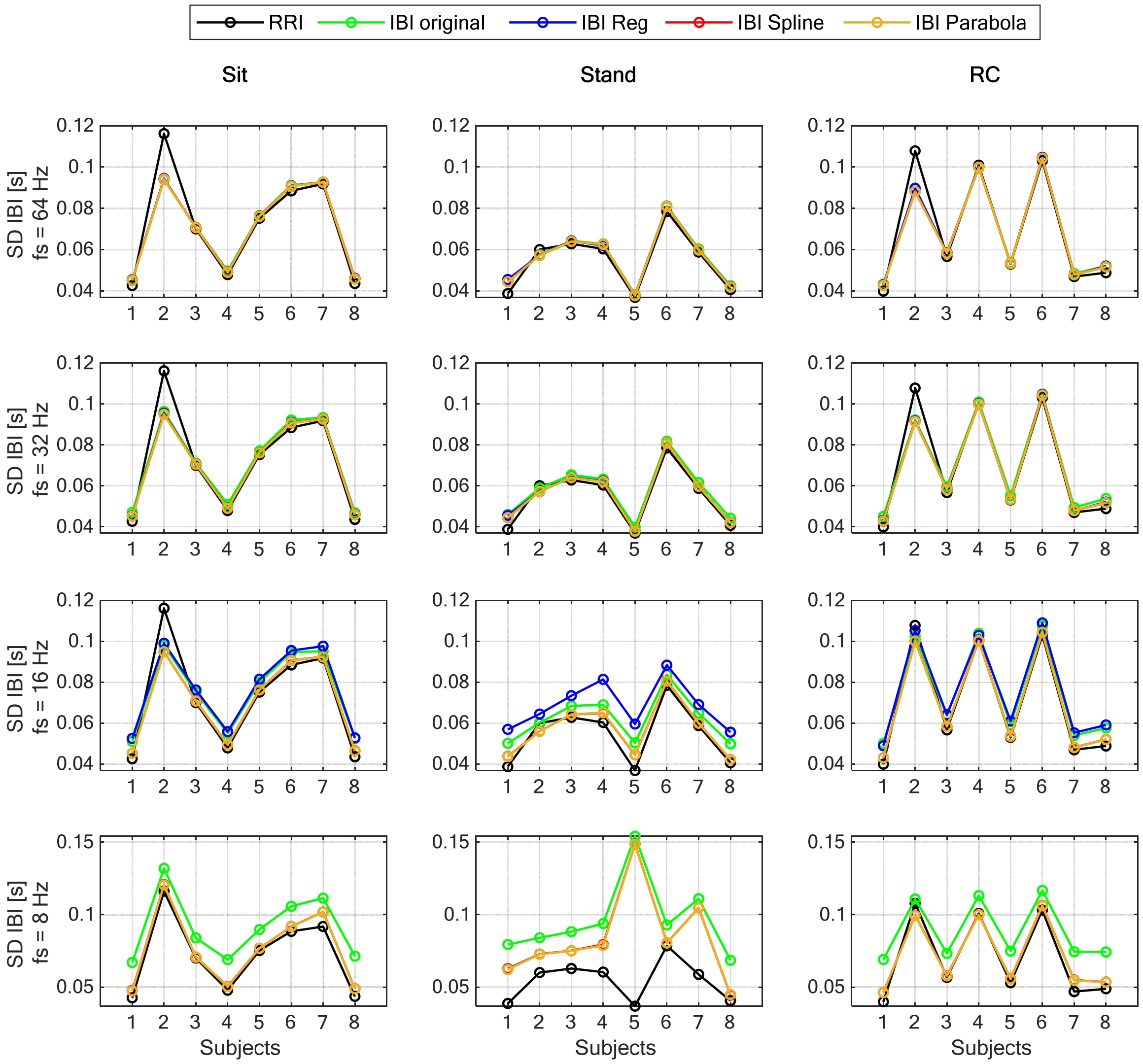

Figure S2. Line charts of the RRI and IBI standard deviation estimated for each subject with the considered PPG interpolation methods, grouped by protocol phase and sampling frequency. As explained in the paper, the REG method could not be applied to PPG signals subsampled at 8 Hz; thus, none of the HRV indices was computed for the REG approach at this frequency.

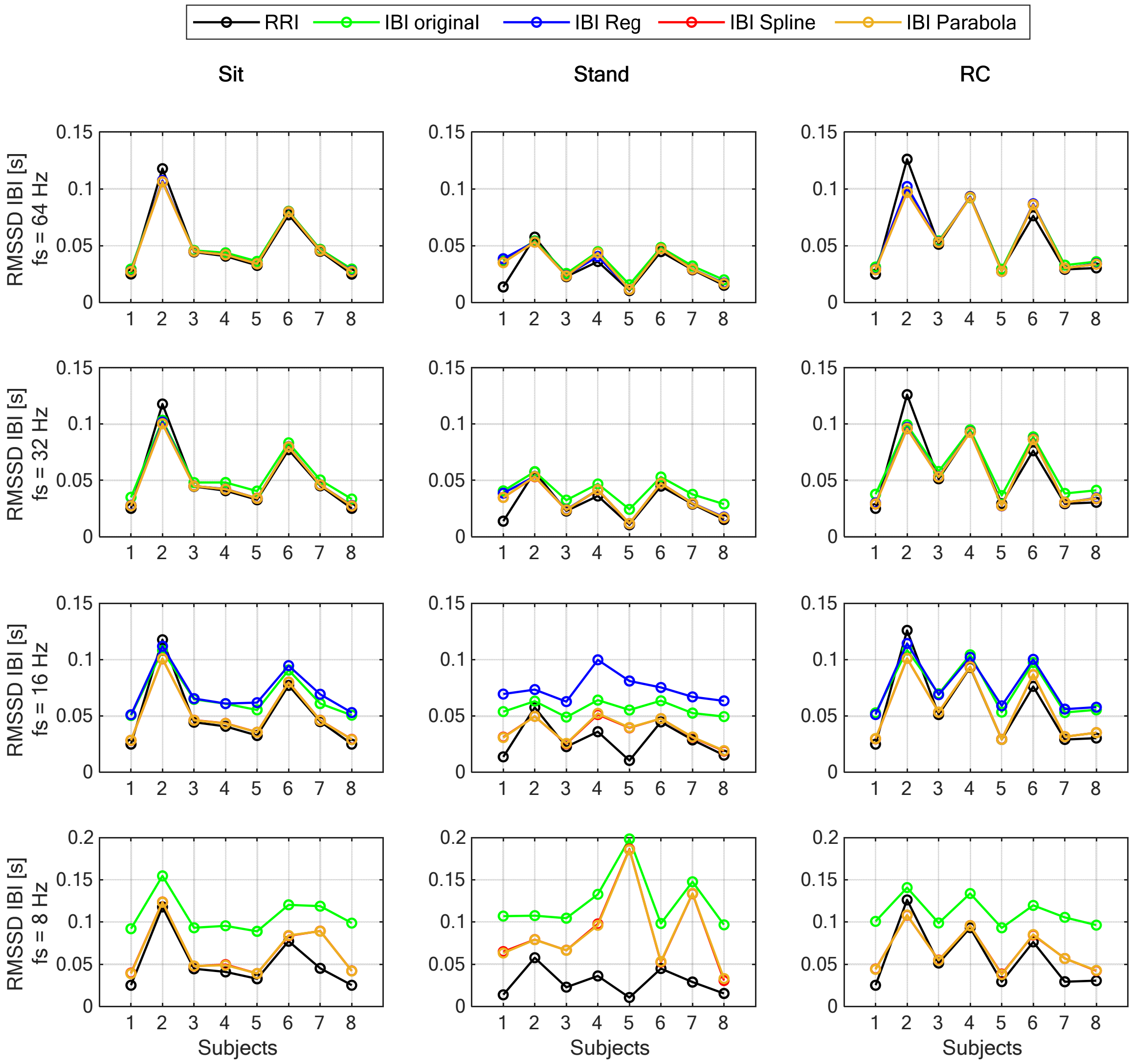

Figure S3. Line charts of the root mean square of successive differences (RMSSD) of the RRIs and IBIs estimated for each subject with the considered PPG interpolation methods, grouped by protocol phase and sampling frequency. As explained in the paper, the REG method could not be applied to PPG signals subsampled at 8 Hz; thus, none of the HRV indices was computed for the REG approach at this frequency.

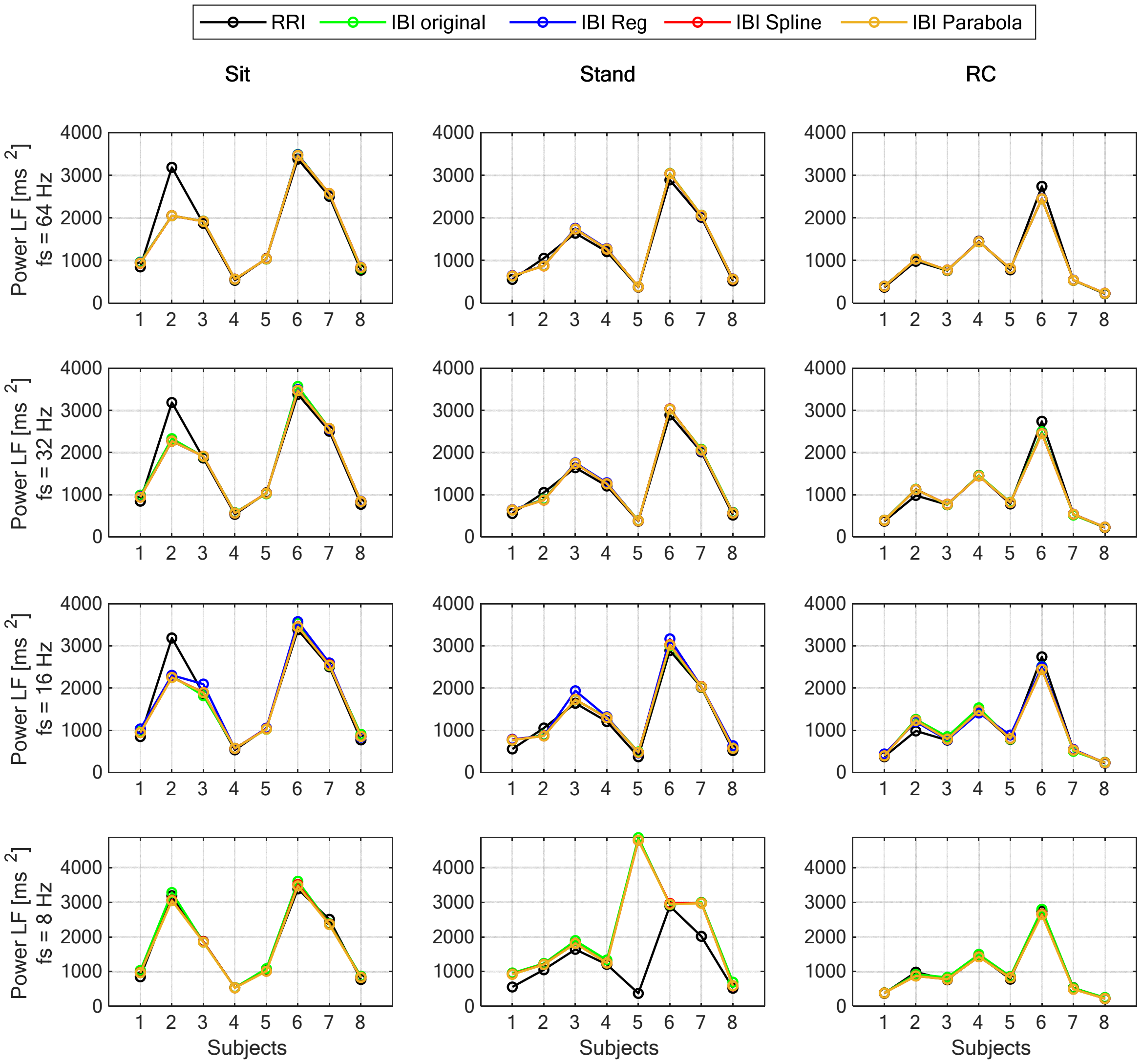

Figure S4. Line charts of the power in the LF band estimated for each subject with the considered PPG interpolation methods, grouped by protocol phase and sampling frequency. As explained in the paper, the REG method could not be applied to PPG signals subsampled at 8 Hz; thus, none of the HRV indices was computed for the REG approach at this frequency.

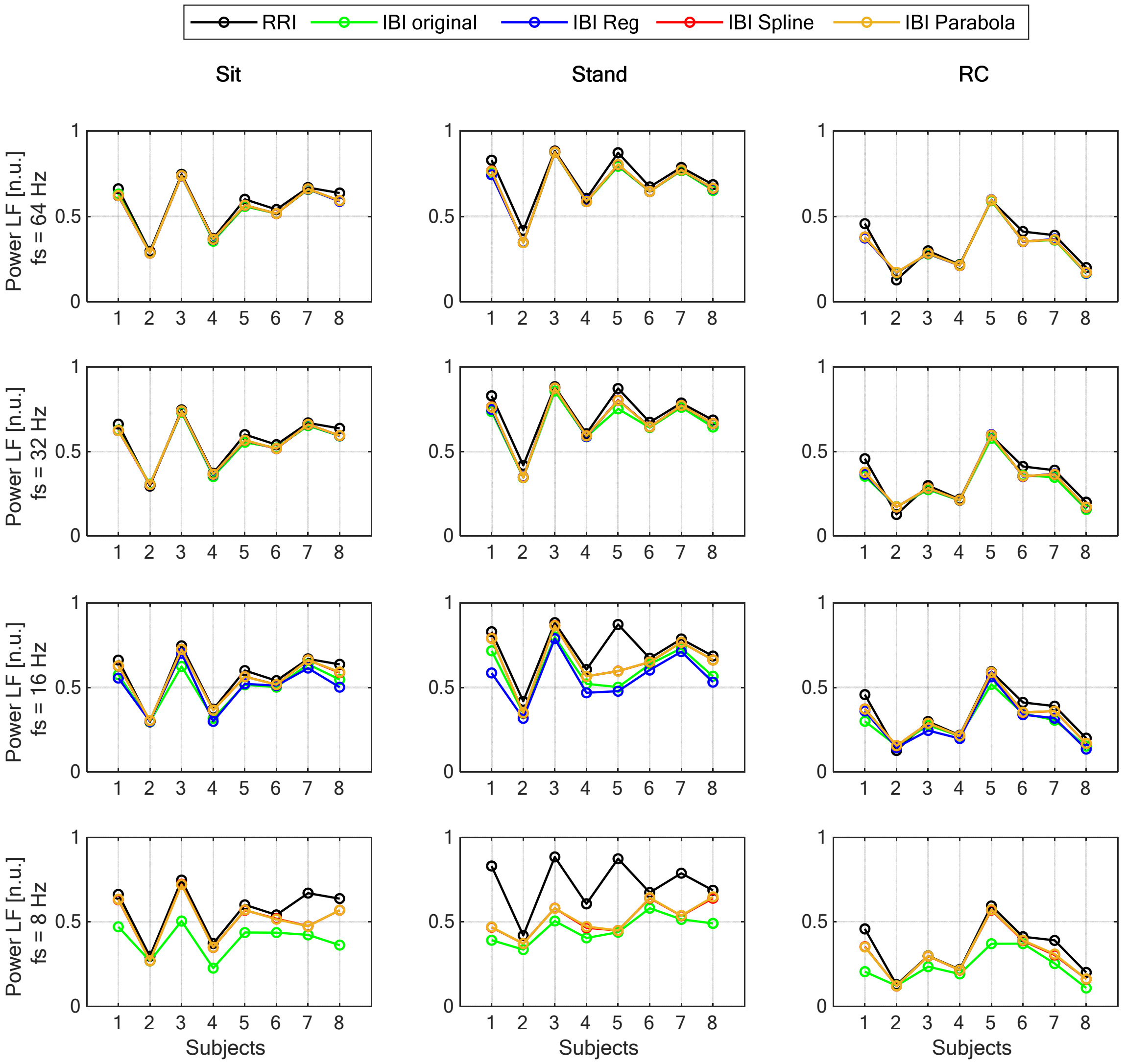

Figure S5. Line charts of the normalized power in the LF band estimated for each subject with the considered PPG interpolation methods, grouped by protocol phase and sampling frequency. As explained in the paper, the REG method could not be applied to PPG signals subsampled at 8 Hz; thus, none of the HRV indices was computed for the REG approach at this frequency.

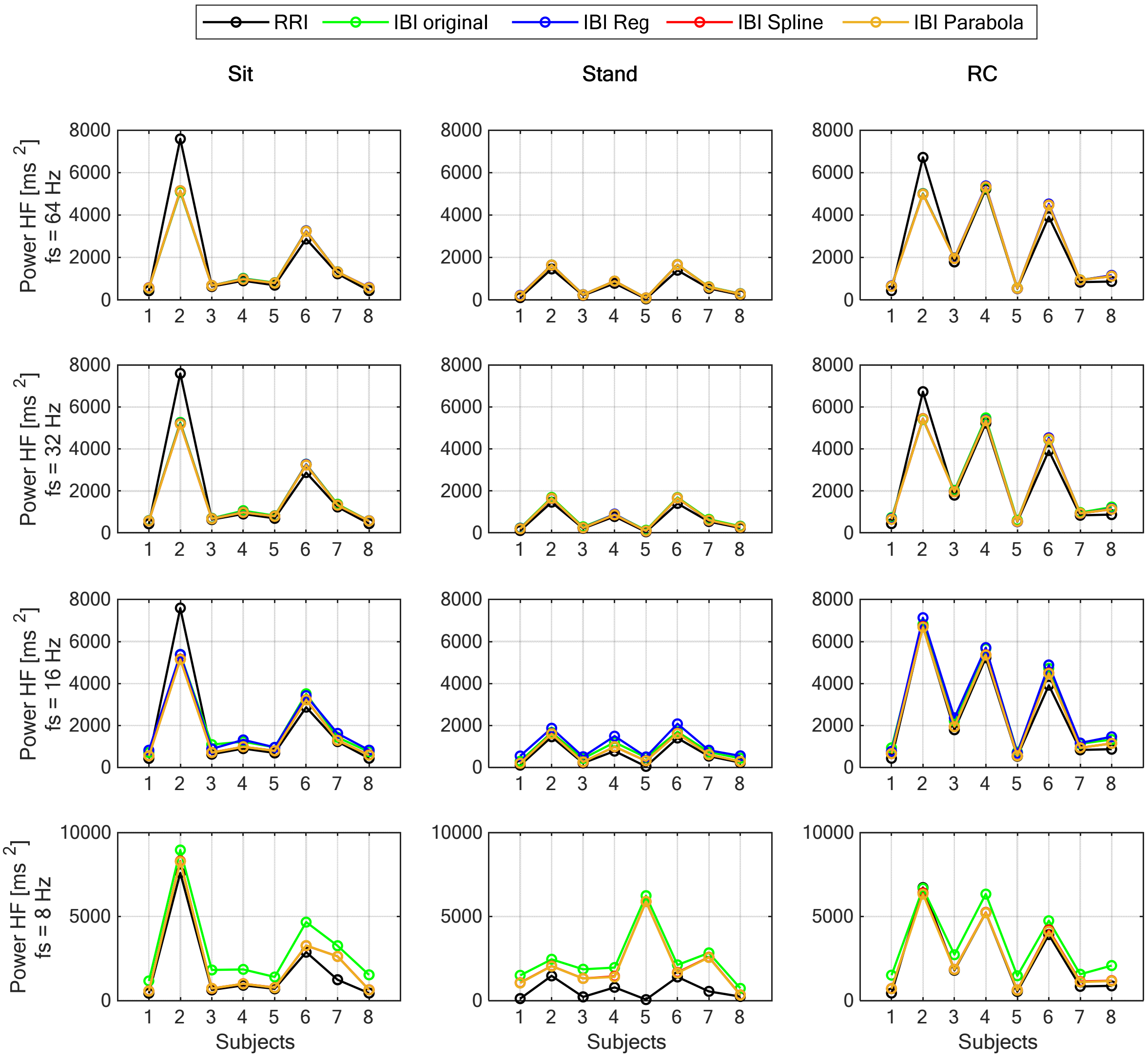

Figure S6. Line charts of the power in the HF band estimated for each subject with the considered PPG interpolation methods, grouped by protocol phase and sampling frequency. As explained in the paper, the REG method could not be applied to PPG signals subsampled at 8 Hz; thus, none of the HRV indices was computed for the REG approach at this frequency.

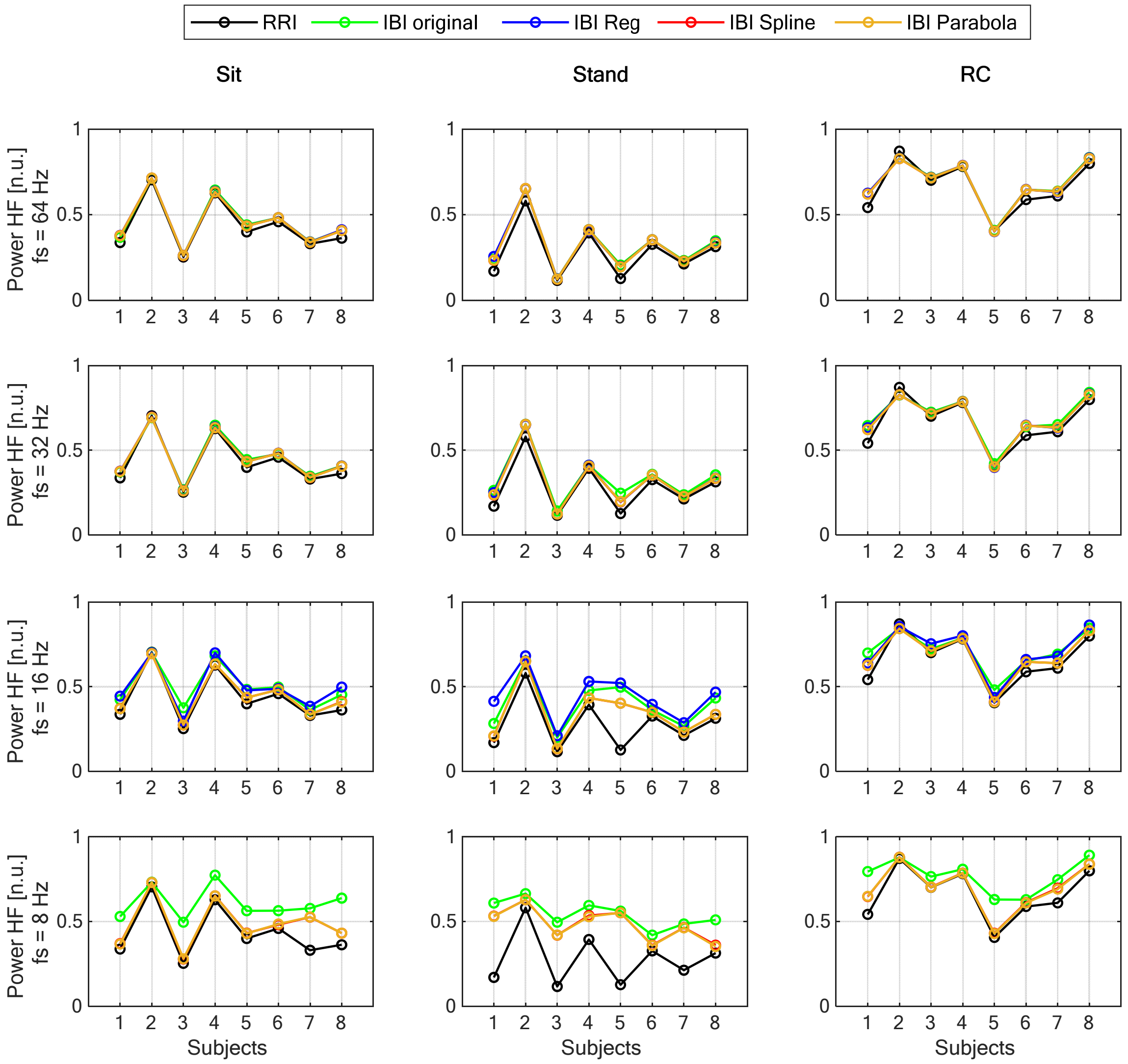

Figure S7. Line charts of the normalized power in the HF band estimated for each subject with the considered PPG interpolation methods, grouped by protocol phase and sampling frequency. As explained in the paper, the REG method could not be applied to PPG signals subsampled at 8 Hz; thus, none of the HRV indices was computed for the REG approach at this frequency.

2. Supplementary Tables

Table S1. FNR, FDR and accuracy observed with each method, grouped by protocol phases. Assessments performed with PPG signals sampled at 32, 16 and 8 Hz.

		FNR (%)		FDR (%)		ACCURACY (%)	
		**SLOPE**	**ENVELOPE**	**SLOPE**	**ENVELOPE**	**SLOPE**	**ENVELOPE**
32 Hz	Sit	0.21	0	0	0.29	99.79	99.71
	Stand	0.57	0	0.04	0.04	99.40	99.96
	CR	0.33	0	0.04	0.25	99.63	99.75
16 Hz	Sit	0.21	0	0	0.29	99.79	99.71
	Stand	0.89	0.04	0	0.07	99.11	99.89
	CR	0.37	0.04	0	0.21	99.63	99.75
8 Hz	Sit	0.83	0.17	0	0	99.17	99.83
	Stand	7.63	1.70	0	0.04	92.37	98.26
	CR	0.83	0.08	0	0.17	99.17	99.75

Table S2. Median absolute error (MAE) and error interquartile range (IQR) of the time-domain HRV indices estimated for each PPG sampling frequency (FS), interpolation method (REG, SPLINE, PARABOLA), and protocol condition (Sit, Stand, CR). ORIGINAL rows refer to the indices estimated from non-interpolated PPG signals.

		Mean IBI (ms)	SD IBI (ms)	RMSSD (ms)	Mean IBI (ms)	SD IBI (ms)	RMSSD (ms)
		MAE ± IQR	MAE ± IQR	MAE ± IQR	MAE ± IQR	MAE ± IQR	MAE ± IQR
			PPG FS = 64 Hz			PPG FS = 16 Hz	
Sit	ORIGINAL	0.31 ± 0.32	2.07 ± 1.72	3.36 ± 2.07	0.42 ± 0.30	6.70 ± 3.78	20.24 ± 9.24
	REG	0.33 ± 0.35	1.88 ± 1.77	2.01 ± 1.54	0.29 ± 0.32	7.51 ± 3.30	22.63 ± 8.15
	SPLINE	0.33 ± 0.35	1.68 ± 1.49[a]	1.84 ± 1.43	0.31 ± 0.36	1.90 ± 1.83	2.88 ± 1.57
	PARABOLA	0.33 ± 0.35	1.69 ± 1.50[a]	1.81 ± 1.41[a]	0.30 ± 0.35	1.85 ± 1.79	2.76 ± 1.60
Stand	ORIGINAL	0.31 ± 0.65	2.14 ± 1.04	4.21 ± 3.63	0.50 ± 1.16	7.37 ± 4.96	27.19 ± 15.94
	REG	0.22 ± 0.67	1.58 ± 1.39	2.21 ± 3.31	0.46 ± 1.19	12.78 ± 9.54	44.13 ± 25.42
	SPLINE	0.22 ± 0.67	1.72 ± 1.52	1.93 ± 5.15	0.53 ± 1.21	3.10 ± 3.32	5.77 ± 13.65
	PARABOLA	0.22 ± 0.67	1.73 ± 1.52	1.91 ± 5.17[a]	0.53 ± 1.22	3.10 ± 3.32	5.96 ± 13.77
CR	ORIGINAL	0.22 ± 3.29	1.70 ± 2.15	4.65 ± 6.14	0.29 ± 2.61	6.58 ± 3.01	22.60 ± 6.76
	REG	0.31 ± 3.31	1.67 ± 2.32	2.93 ± 6.06	0.51 ± 2.56	7.71 ± 4.45	25.19 ± 12.55
	SPLINE	0.32 ± 3.29	1.38 ± 1.80	2.54 ± 5.45	0.34 ± 2.58	1.53 ± 2.29	3.62 ± 6.30
	PARABOLA	0.32 ± 3.29	1.37 ± 1.81	2.57 ± 5.46	0.34 ± 2.58	1.48 ± 2.34	3.65 ± 6.42
			PPG FS = 32 Hz			PPG FS = 8 Hz	
Sit	ORIGINAL	0.37 ± 0.27	3.14 ± 2.35	7.46 ± 3.41	0.37 ± 1.55	18.44 ± 7.59	55.40 ± 24.42
	REG	0.33 ± 0.35	1.89 ± 1.81	2.04 ± 1.60	-	-	-
	SPLINE	0.33 ± 0.35	1.68 ± 1.53[a]	1.75 ± 1.40[a]	0.50 ± 1.37	3.92 ± 3.09	7.54 ± 9.71[a]
	PARABOLA	0.33 ± 0.35	1.70 ± 1.55[a]	1.74 ± 1.38[a]	0.50 ± 1.37	3.74 ± 2.97[a]	7.37 ± 9.43[a]
Stand	ORIGINAL	0.28 ± 0.60	2.93 ± 0.76	10.20 ± 5.26	2.75 ± 11.36	30.70 ± 21.70	87.46 ± 40.34
	REG	0.21 ± 0.67	1.73 ± 1.43	2.33 ± 3.81	-	-	-
	SPLINE	0.22 ± 0.66	1.46 ± 1.52	2.05 ± 3.94[a]	2.78 ± 11.24	15.91 ± 26.91	47.44 ± 64.75[a]
	PARABOLA	0.22 ± 0.66	1.51 ± 1.46	2.10 ± 3.92	2.78 ± 11.24	15.47 ± 26.45[a]	46.57 ± 62.94[a]
CR	ORIGINAL	0.24 ± 3.31	2.65 ± 3.13	9.94 ± 5.56	0.57 ± 1.92	19.19 ± 13.51	55.63 ± 28.87
	REG	0.31 ± 3.31	1.61 ± 2.49	3.00 ± 6.16	-	-	-
	SPLINE	0.32 ± 3.30	1.42 ± 1.90	2.56 ± 5.59	0.49 ± 1.98	3.98 ± 5.17	10.43 ± 12.74
	PARABOLA	0.32 ± 3.30	1.36 ± 1.96	2.60 ± 5.55[a]	0.49 ± 1.97	3.81 ± 5.17[a]	10.52 ± 12.59[a]

a. Significantly different from ORIGINAL (Bonferroni-corrected comparisons, $p<0.05$), with effect size (r)>0.5.

Table S3. Median absolute error (MAE) and error interquartile range (IQR) of the frequency-domain HRV indices estimated for each PPG sampling frequency (FS), interpolation method (REG, SPLINE, PARABOLA), and protocol condition (Sit, Stand, CR). ORIGINAL rows refer to the indices estimated from non-interpolated PPG signals. Since absolute errors computed from the normalized LF and HF powers (i.e., *Power LF (n.u)* and *Power HF (n.u.)*) are equal by definition, their MAE and IQR are shown in the same table column.

		Power LF (ms^2)	Power HF (ms^2)	Power LF or HF (n.u.)	Power LF (ms^2)	Power HF (ms^2)	Power LF or HF (n.u.)
		**MAE ± IQR**	**MAE ± IQR**	**MAE ± IQR**	**MAE ± IQR**	**MAE ± IQR**	**MAE ± IQR**
		**PPG FS = 64 Hz**			**PPG FS = 16 Hz**		
Sit	ORIGINAL	53.8 ± 73.3	124.6 ± 155.2	0.020 ± 0.026	85.8 ± 111.0	319.8 ± 275.9	0.069 ± 0.053
	REG	63.6 ± 61.0	123.6 ± 183.0	0.017 ± 0.029	137.3 ± 164.3	392.6 ± 154.3	0.064 ± 0.056
	SPLINE	65.9 ± 46.5	118.9 ± 176.1	0.017 ± 0.029	58.9 ± 59.4	114.7 ± 176.0	0.022 ± 0.028
	PARABOLA	65.4 ± 48.2	117.3 ± 172.5	0.017 ± 0.028	53.6 ± 62.6	111.6 ± 166.8[a]	0.022 ± 0.027
Stand	ORIGINAL	69.6 ± 84.2	83.3 ± 94.9	0.031 ± 0.051	108.8 ± 72.6	212.1 ± 162.5	0.085 ± 0.053
	REG	81.9 ± 88.7	76.8 ± 98.7	0.026 ± 0.056	148.8 ± 150.0	425.5 ± 254.6	0.119 ± 0.114
	SPLINE	71.3 ± 77.7	71.5 ± 102.8[a]	0.024 ± 0.049	96.5 ± 93.3	120.4 ± 154.0	0.031 ± 0.033
	PARABOLA	71.6 ± 77.9	71.8 ± 102.0[a]	0.024 ± 0.050	96.4 ± 89.9	114.6 ± 150.4	0.032 ± 0.033
CR	ORIGINAL	21.7 ± 28.3	204.0 ± 335.5	0.032 ± 0.039	55.1 ± 131.4	426.2 ± 232.8	0.056 ± 0.054
	REG	20.2 ± 23.7	204.2 ± 327.6	0.027 ± 0.040	54.5 ± 150.7	445.6 ± 251.5	0.059 ± 0.047
	SPLINE	18.7 ± 28.5	184.3 ± 300.5	0.028 ± 0.041	22.6 ± 128.0	148.1 ± 184.1	0.029 ± 0.038[a]
	PARABOLA	18.2 ± 27.9	182.2 ± 300.4	0.027 ± 0.041	21.2 ± 125.0	137.3 ± 177.1[a]	0.029 ± 0.038[a]
		**PPG FS = 32 Hz**			**PPG FS = 8 Hz**		
Sit	ORIGINAL	66.2 ± 125.2	146.9 ± 152.2	0.019 ± 0.024	92.1 ± 123.9	1136.1 ± 735.7	0.178 ± 0.120
	REG	61.7 ± 72.8	119.6 ± 180.5	0.017 ± 0.027	-	-	-
	SPLINE	66.0 ± 55.6	116.0 ± 171.0	0.016 ± 0.028	95.4 ± 107.4	167.2 ± 459.0[a]	0.029 ± 0.026[a]
	PARABOLA	66.0 ± 57.6	113.5 ± 168.0[a]	0.016 ± 0.027	71.8 ± 98.9	156.6 ± 447.3[a]	0.028 ± 0.023[a]
Stand	ORIGINAL	75.0 ± 59.9	106.2 ± 94.0	0.036 ± 0.058	216.8 ± 542.0	1282.3 ± 1113.7	0.237 ± 0.263
	REG	84.1 ± 88.8	79.3 ± 96.7[a]	0.025 ± 0.052[a]	-	-	-
	SPLINE	68.2 ± 76.1	70.6 ± 90.5[a]	0.024 ± 0.054[a]	165.0 ± 593.4	800.8 ± 1136.8	0.197 ± 0.285[a]
	PARABOLA	68.0 ± 75.9	71.8 ± 89.8[a]	0.024 ± 0.055	159.7 ± 599.5[a]	770.7 ± 1150.7[a]	0.193 ± 0.289[a]
CR	ORIGINAL	22.4 ± 86.4	269.7 ± 259.9	0.043 ± 0.029	47.5 ± 42.3	932.4 ± 312.2	0.078 ± 0.147
	REG	18.2 ± 75.9	206.9 ± 333.4	0.027 ± 0.040	-	-	-
	SPLINE	17.6 ± 76.6	185.1 ± 296.0	0.027 ± 0.041	36.9 ± 48.5	273.6 ± 224.6	0.024 ± 0.057
	PARABOLA	17.7 ± 77.7	183.7 ± 296.4[a]	0.027 ± 0.041[a]	33.6 ± 62.0	229.9 ± 214.0[a]	0.022 ± 0.055[a]

a. Significantly different from ORIGINAL (Bonferroni-corrected comparisons, $p<0.05$), with effect size (r)>0.5

© 2022 by the authors. Licensee MDPI, Basel, Switzerland. This article is an open access article distributed under the terms and conditions of the Creative Commons Attribution (CC BY) license (https://creativecommons.org/licenses/by/4.0/).
